# Commercially available bioinks and state-of-the-art lab-made formulations for bone tissue engineering: A comprehensive review

**DOI:** 10.1016/j.mtbio.2024.101341

**Published:** 2024-11-14

**Authors:** Elena Alina Chiticaru, Mariana Ioniță

**Affiliations:** a Faculty of Medical Engineering, National University of Science and Technology Politehnica Bucharest, Gh Polizu 1-7, 011061, Bucharest, Romania; b Advanced Polymer Materials Group, National University of Science and Technology Politehnica Bucharest, Gh Polizu 1-7, 011061, Bucharest, Romania

**Keywords:** Bone tissue engineering, Bioprinting, Commercial bioink, Printable formulation

## Abstract

Bioprinting and bioinks are two of the game changers in bone tissue engineering. This review presents different bioprinting technologies including extrusion-based, inkjet-based, laser-assisted, light-based, and hybrid technologies with their own strengths and weaknesses. This review will aid researchers in the selection and assessment of the bioink; the discussion ranges from commercially available bioinks to custom lab-made formulations mainly based on natural polymers, such as agarose, alginate, gelatin, collagen, and chitosan, designed for bone tissue engineering. The review is centered on technological advancements and increasing clinical demand within the rapidly growing bioprinting market. From this point of view, 4D, 5D, and 6D printing technologies promise a future where unprecedented levels of innovation will be involved in fabrication processes leading to more dynamic multifunctionalities of bioprinted constructs. Further advances in bioprinting technology, such as hybrid bioprinting methods are covered, with the promise to meet personalized medicine goals while advancing patient outcomes for bone tissues engineering applications.

## Graphical abstract

Schematic representation for the design of commercial and lab-made bioinks for 3D bioprinting with applications in bone tissue engineering. Created with BioRender.com.

**Figure:**
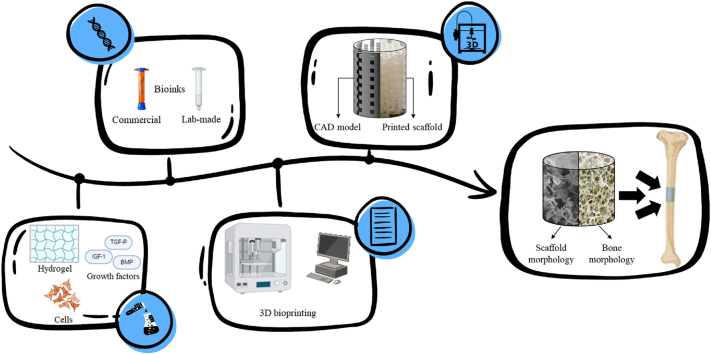

## 1 Introduction

The advancement of three-dimensional (3D) bioprinting has brought about a new era in regenerative medicine and opened up unprecedented opportunities for tissue engineering and personalized healthcare. Among the many areas of utilization, bone tissue engineering (BTE) is one of the most important fields that can benefit from this technology. The potential power of 3D bioprinting lies in its ability to create complex three-dimensional structures with high precision using bioinks (i.e., a mixture of cells, growth factors, and biomaterials [1]) that are tailor-made to resemble extracellular matrices (ECMs) found in natural tissues at the site being treated [2].

Traditional bone grafts, such as autografts (currently used as the gold standard for treating bone defects) and allografts, have been the main solutions for bone repair [[3], [4], [5]]. Recently, there has been a shift toward the advancement of biomaterials and more sophisticated solutions that better mimic native tissues.

Conversely, designing solutions for bone tissue repair is not straightforward due to the complex bone structure and functions, i.e., protects organs, produces blood cells, stores minerals, and provides mechanical support. Regarding the structure, 80 % of total bone mass is contributed by cortical bone, which is dense and strong, and 20 % by trabecular bone, which is porous with large surface-to-volume ratio. From a chemical structure point of view, bones are composed of lamellae of mineralized collagen fibers, with hydroxyapatite (HAP) and collagen type I (COL-I) as their main components. Bone tissue is formed, maintained, and permanently remodeled by osteoblasts, osteocytes, and osteoclasts [6].

To effectively replicate bone tissue, several key features must be addressed. As aforementioned, bones endure major stresses, therefore mechanical properties such as compressive strength (0.1–0.4 GPa for trabecular bone and 14.1–27.6 GPa for cortical bone [7]) and tensile strength (10.4 ± 3.5 GPa for trabecular bone and 7.1–24.5 GPa for cortical bone [7]) are critical properties to be considered. Secondly, but equally important, the engineered tissue must have the ability to degrade with a similar rate as the new tissue is growing, and to support osteogenesis, mineralization, and vascularization, in order to develop functional tissue [6,8].

Anatomically accurate bone tissue, containing intricate internal architectures that imitate natural porosity and possess appropriate mechanical properties, can be created using the additive manufacturing technology, specifically the bioprinting technique [9,10]. Moreover, 3D bioprinting makes it possible to customize implants according to the individual's needs [11,12], especially with the aid of artificial intelligence (AI) [13], thereby reducing the risk of complications and offering more promising results.

Furthermore, the utilization of bioinks allows for the incorporation of multiple cell types, growth factors, and other bioactive molecules into the printed constructs [[14], [15], [16]]. Such a multimodal approach is believed to create a supportive environment for bone formation, leading to accelerated healing and improved outcomes.

However, as with any emerging field, there are certain obstacles that the 3D bioprinting of BTE faces. The first major challenges are represented by the production and printability of appropriate bioinks to address the mechanical and biological characteristics of bone tissue [17,18]. Such formulations must be easily printable, biocompatible, and biodegradable to a certain extent [19], and should be able to offer the required mechanical support [20]. Moreover, controlling the biological functions of the material still poses a significant challenge, especially in terms of maintaining cell viability during the bioprinting process [21]. Ensuring the vascularization of the printed scaffolds is another critical obstacle, since sufficient blood flow is necessary for the designed bone tissue to integrate and survive [22].

There is a wide variety of materials and approaches used in the development of bioinks for BTE, primarily guided by the goal of biomimicry, aiming to replicate the structural complexity of bone tissue. In general, lab-made bioinks are based on polysaccharides and proteins, such as alginate (Alg), agarose (Aga), chitosan (Chi), collagen (Col), and gelatin (Gel), which form a polymeric network that serves as the basis for mimicking the organic structure of bone [23]. Additionally, nanomaterials like HAP and other minerals are incorporated to enhance the mechanical properties of the bioinks, as they replicate the inorganic components of bone tissue [24]. Moreover, decellularized extracellular matrix (dECM) is considered a game changer in the field of BTE, receiving significant interest because of its ability to mimic the native microenvironment and exert tissue-specific functions. dECM is produced by removing cellular components from tissues, while keeping essential bioactive molecules including proteins, growth factors, and collagen. It has been shown that dECM bioactive elements play a critical role in regulating cell adhesion, proliferation, and differentiation [25].

An important outcome of major advancements in lab-made (bio)ink formulations is that commercially available inks and bioinks have begun to establish their presence on the market, offering effective, reproducible, and convenient solutions for researchers and medical professionals. Currently, some of the companies taking the lead in this area include CELLINK and Advanced Biomatrix (part of BICO group), REGENHU, and TissueLabs, which produce various types of printable mixtures, specifically designed for certain tissues. The main aim behind these products is to enhance reproducibility, usability, and compatibility with different bioprinters so as to expedite the translation of 3D printing from bench-top experimentation into clinical application [26]. Even though substantial progress has been made in the bioprinting field, continuous research and development still play a vital role in overcoming the challenges faced at present and capitalizing on the potential that this innovative approach offers. Future progress in BTE will provide personalized treatments with increased effectiveness and accessibility, improving the quality of life for millions of patients around the world.

In the scientific literature, there are several valuable review articles discussing the advancement of bioprinting technologies in BTE [[27], [28], [29]] and the development of various printable formulations. Notably, Dobrisan et al. [30] examine the progress of gelatin methacryloyl (GelMA)-based inks specifically designed for BTE applications, along with a comparison of photoinitiators commonly used and the influence of 3D printing technology. Similarly, Cernencu et al. [31] provide a detailed review of inks derived from gellan gum and the methods to optimize their properties, while Tolmacheva et al. [32] discuss the utilization and importance of various calcium phosphates (CaP) in the fabrication of printable formulations for bone regeneration applications. Additionally, Kaith et al. [33] focus on the polysaccharide-based inks designed for the treatment of bone injuries and their potential to be used in clinical settings.

In contrast to the previous reviews, which focused on formulations based on specific materials with broader applicability [30,31], this review presents an exhaustive and up-to-date analysis (from the last 3–4 years) of both commercial and lab-made (bio)ink formulations specifically designed for BTE applications. The scope of this review is broader, aiming to provide a comprehensive overview of the latest developments in printable formulations, making it distinct from the material-specific approaches of our earlier works. As this is a field where research evolves rapidly, new advancements are continually emerging.

Therefore, as mentioned above, in this review article, we present a comprehensive overview of bioprinting technologies and (bio)inks for BTE applications. Particularly, the first chapter describes different bioprinting technologies, including extrusion-based, inkjet-based, laser-assisted, light-based, and hybrid methods, in terms of working principle, advantages, and limitations. Moreover, a dedicated subchapter addresses the mutual influence between bioprinting technologies and bioink formulations. In the next chapter, printable formulations are categorized and examined, differentiating between commercially available (bio)inks and innovative lab-made printable formulations, particularly those based on natural polymers like agarose, alginate, gelatin, collagen, and chitosan, each of them studied for their suitability in BTE applications. The following chapter describes the key challenges in this field, such as regulatory and technical provocations, and discusses future directions, including 4D, 5D, and 6D bioprinting, which promise to revolutionize the fabrication processes by enabling dynamic, multifunctional constructs. In the last chapter we conclude with some final insights on how bioprinting is enabling personalized medicine and enhances patient outcomes in regenerative orthopedics.

## 2 Bioprinting methods

**Fig. 1:**
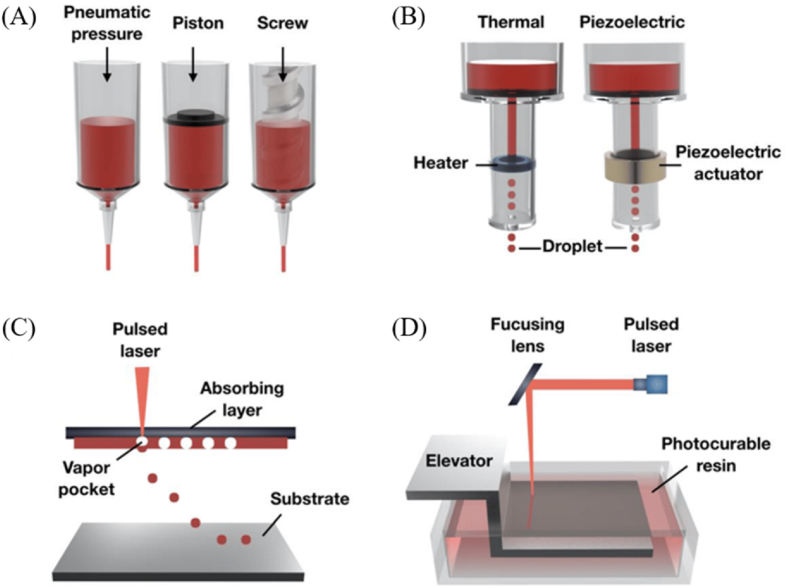
Schematic illustration of the (a) extrusion-based, (b) inkjet-based, (c) laser-assisted, and (d) light-based (stereolithography) 3D bioprinting systems [35] (CC BY 4.0).

Additive manufacturing can be categorized into four primary types based on the 3D printing technique: extrusion-based, inkjet based, laser-assisted, and light-based (Fig. 1). Nowadays, the medical sector and the manufacturing of biomaterials have undergone significant changes as a result of the fast rise of 3D bioprinting in the medical area. It has been possible to create complex tissues and organs with this technology, and, in order to achieve the desired scaffold architecture, these methods are applied either alone or in combination [34].

### 2.1 Extrusion-based bioprinting

Based on additive manufacturing principles, extrusion-based printing has the potential to produce complex and functional three-dimensional tissue constructs. This method is based on the carefully regulated application of inks or bioinks via nozzles, which permits the layer-by-layer assembly of the desired scaffold [36]. The capacity of extrusion-based printing to work with a range of printable formulations, such as hydrogels [37] and cell-rich matrices [38], accounts for its adaptability and makes it promising for different fields of tissue regeneration.

Typically, an automated three-axis robotic system plus a dispensing head constitute an extrusion-based 3D printer [39]. The dispensing head, which is computer controlled, applies the ink onto a stage by either moving along the three axes or remaining stationary as the stage moves [40]. Because it produces better structural integrity by precisely and continuously depositing filaments rather than liquid droplets, this printing technique is frequently chosen [38]. The printing resolution of this technique is relatively low, in the range of 100–500 μm [41], and it is influenced mainly by the pressure (10–500 kPa) [42,43], nozzle size (14 gauge – 34 gauge) [44,45], speed (0.2–18 mm/s) [46,47], and ink viscosity [48]. The mechanism of operation for most extrusion-based printers is the same: inks are fed into a syringe barrel and extruded through a micro-nozzle tip. Multiple print heads on these printers allow different inks to be used simultaneously in a single construction and supporting materials that aid in preserving the structural integrity of the construct during printing can be employed [38,40]. Setting up parameters like speed, pressure, temperature, and the distance between the stage and the tip is essential before the printing process begins. Based on the dispensing mechanism, extrusion-based bioprinting techniques can be broadly classified into three types: pneumatic, screw-based, and piston-based systems (Fig. 1 A).

Extrusion-based printing methods encompass a wide range of approaches, each customized for particular tissue engineering purposes. With direct extrusion, inks are carefully deposited onto a substrate layer-by-layer, enabling the exact building of intricate tissue architectures [49]. Moreover, in order to expedite the solidification of the printed object, the ink can be simultaneously deposited and exposed to a coagulating agent during extrusion in a coagulation bath [50]. This method is particularly useful for supporting the construction of delicate or complex designs and improving structural integrity [51]. Extrusion in a support bath uses during the printing process a sacrificial substance as a temporary scaffold [52], facilitating the creation of intricate, self-supporting structures [53]. The last method is co-axial extrusion, which allows core-shell structures to be created by simultaneously extruding two materials through concentric nozzles [54]. Together, these extrusion-based methods increase the adaptability and accuracy of 3D bioprinting and provide a range of instruments for researchers to tackle the many difficulties associated with tissue creation.

Moreover, high-throughput extrusion-based printers have been developed as a result of initiatives to increase printing speed and scalability [[55], [56], [57]]. These developments not only speed up the manufacturing process but also pave the way for the application of extrusion-based bioprinting technologies in clinical settings. However, this method is not without challenges despite its adaptability. The potential shear stress that cells may endure during the extrusion process is one major issue since it might have an impact on their survival and functionality [38,58]. Researchers are presently exploring strategies to reduce shear stress by optimizing the characteristics of bioinks and printing conditions [59] and attaining the accurate spatial distribution of various cell types inside a construct is still challenging [60]. Ongoing research in this area is focused on addressing concerns about cross-contamination between different bioinks [61] and improving nozzle design [62,63].

The future of extrusion-based printing holds exciting possibilities, considering that it is anticipated that integration with cutting-edge imaging methods and real-time monitoring systems would improve printing process accuracy and precision [64]. Furthermore, real-time printing parameter optimization is possible with the integration of AI and machine learning algorithms [65,66], which enhances the repeatability and dependability of extrusion-based printing. This method is likely to become more widely applicable in the future as new biomaterials and bioink formulations are continuously explored, which will allow for the creation of more complex tissues with improved physiological relevance.

As a flexible and scalable method for tissue engineering, extrusion-based bioprinting has become a major force in the 3D bioprinting area, holding immense promise to transform personalized healthcare and regenerative medicine as researchers explore its complexities, address various challenges, and capitalize on breakthroughs. This method is at the forefront of innovation in the quest to regenerate functioning tissues and organs because of its capacity to accurately create complex tissue structures.

### 2.2 Inkjet-based bioprinting

Inkjet-based bioprinting, inspired by conventional desktop inkjet printing [67], uses print heads to precisely eject droplets, which are then used to form complex patterns of bioinks [68]. This method can be classified into thermal and piezoelectric inkjet bioprinting (Fig. 1 B) and represents a useful tool in the effort to replicate complex biomimetic structures, since it enables high-throughput production with remarkable resolution [69] and spatial control of cell differentiation [70].

The precise deposition of tiny droplets of bioink onto a substrate is the fundamental principle of inkjet-based bioprinting [69]. Layer-by-layer building of complex tissue assemblies is made possible by the fine control of droplet size, velocity, and location, which is achieved by the use of print heads fitted with nozzles [71,72]. The possibility of contamination, ink waste, and damaging cells during the printing process is reduced by the drop-on-demand and non-contact nature of inkjet-based bioprinting [48,73,74]. The printing resolution of this technique is lower compared to extrusion-based bioprinting, reaching 50 μm [48], and it is influenced mainly by the surface contact angle [41].

In order to improve cell viability and build accuracy, recent developments in inkjet-based bioprinting have concentrated on improving printing conditions [75]. The range of applications has broadened through the incorporation of diverse cell types and biomaterials, enabled by advancements in nozzle designs, ink formulations, and printing techniques. Systems with several materials and nozzles may now be used to deposit various bioinks simultaneously, allowing for the production of heterogeneous tissue constructs with a variety of cell populations [[76], [77], [78]]. Furthermore, the exact positioning of bioinks was made possible by developments in droplet control technologies, which favored the design of complex scaffolds with high resolution [48,79,80]. Systems for real-time feedback and monitoring are also being incorporated to improve repeatability and guarantee printing accuracy [[81], [82], [83]].

Although inkjet-based bioprinting provides very accurate results, there are still some limitations to consider. The possible effect of droplet production and ejection on cell viability is one major cause for concern [84]. To reduce shear stress and preserve cell integrity throughout the printing process, researchers are actively investigating methods to improve the qualities of bioink, such as composition and viscosity [85]. However, it is still difficult to distribute cells uniformly within printed structures, especially when dealing with highly populated tissues [86,87]. Researchers are focusing their efforts to increase the homogeneity of cell distribution inside the printed structures and to solve difficulties related to cell clustering [88].

Inkjet-based bioprinting has promising future potential and it is anticipated that additional developments in bioink formulations, as well as in nozzle and droplet control technology, will enhance the accuracy and potential of this method. Enhancement of inkjet-based bioprinting's reliability and repeatability can be achieved by integration with sophisticated imaging modalities and real-time monitoring systems [81,82]. The development of bioink formulations that react to environmental cues, including light or temperature, offers a way to increase the adaptability of bioprinting using inkjet technology [48,89]. Furthermore, real-time parameter optimization, problem-solving, and performance enhancement are all possible with the integration of AI for adaptive control of the printing process [82,90].

Bioprinting using inkjet technology has become a potent and accurate method for creating biomimetic tissue architectures. It is an important tool in the field of tissue engineering and regenerative medicine because of its high resolution, scalability, and adaptability. Inkjet-based bioprinting is set to play a key role in advancing the field as scientists around the world focus on overcoming obstacles and finding new ways to improve this technology, which ultimately will lead to the realization of individualized and functional tissue structures for therapeutic purposes.

### 2.3 Laser-assisted bioprinting

Laser-assisted bioprinting (LAB) represents a non-contact approach to bioprinting, relying on the controlled use of laser energy to propel bioinks onto a substrate [91]. This method provides a potential path toward simulating the complexities of natural tissues by enabling the construction of elaborate tissue architectures with high accuracy [92].

The main principle behind LAB is represented by the creation of a laser-induced microbubble inside an absorbent layer of the bioink (Fig. 1 C), using lenses and mirrors to direct the laser beam [91]. This microbubble expands rapidly, generating a pressure wave that precisely directs bioink droplets onto the substrate [93]. This technique reduces the possibility of cellular harm by assembling complex tissue structures layer-by-layer without physical touch due to its accuracy and speed [94] and high-viscous bioinks can be used due to this method not requiring the use of nozzles. Moreover, LAB is the most precise bioprinting method and has the highest printing resolution, in the range of 1–10 μm [95].

The main goals of the latest developments in LAB have been to increase its applicability and improve its capabilities. The creation of hybrid systems integrates LAB with other bioprinting techniques for increased adaptability, while the integration of several bioinks enables the construction of heterogeneous tissue architectures [92]. Scientists are now investigating complex biomaterials, such as bioinks containing certain bioactive elements, to enhance the performance and biocompatibility of printed structures [[96], [97], [98]]. Multiphoton absorption and the use of ultrafast lasers are two examples of how advances in laser technology have improved printing accuracy and resolution [[99], [100], [101]]. Critical feedback can be provided during printing by real-time imaging and monitoring devices, guaranteeing precision and reproduction [102].

LAB also has limitations related to the characteristics of the bioink and laser technology. Ongoing considerations include developing bioinks appropriate for different cell types and optimizing bioink formulations for laser energy compatibility [103,104]. In order to increase the usability of LAB in a variety of tissue engineering applications, these issues must be addressed first. However, exciting opportunities exist for the future of LAB, as continued research is anticipated to improve laser technology and bioink compositions. Incorporating AI to regulate the printing process in real time [105] and integrating it with sophisticated imaging software would likely help LAB overcome its present obstacles and enhance its tissue engineering potential.

In the realm of 3D bioprinting, laser-assisted bioprinting is a groundbreaking method that can provide unmatched printing accuracy. The field of tissue engineering might be completely changed by LAB as scientists work to find new ways to advance the field, making it possible to fabricate complex and functional tissue structures with previously unattainable precision and reproducibility.

### 2.4 Light-based bioprinting

Light-based printing harnesses the controlled application of light to selectively cure inks layer-by-layer, offering a non-contact and high-resolution methodology for the precise construction of complex tissue structures [106]. This technique holds great promise for advancing tissue engineering applications by providing an innovative means to replicate the complexities of native tissues.

Layer-by-layer solidification of inks by photopolymerization induced by light is the basic principle of light-based bioprinting [107]. While digital light processing uses a digital light projector to build patterns and cure particular portions of the bioink [108], stereolithography uses a laser or other light source to selectively cure sections of a liquid resin [109] (Fig. 1 D). The adaptability of this method makes it possible to create structures with outstanding printing resolution [110].

The resolution (25–50 μm) [111], speed (0.66 mm³/s) [112], and adaptability [107] of light-based bioprinting have been recently improved. The range of printed materials has increased due to advancements in photopolymerizable materials, i.e., inks with different mechanical and functional characteristics [113]. The use of several inks and multi-material printing allows for the production of diverse tissue shapes that closely resemble the complex nature of biological tissues [114]. High-speed digital light processing and other developments in light projection technology have drastically shortened printing times without sacrificing accuracy [115], and the printing process has been made more accurate and reproducible by integrating monitoring tools and machine learning [116].

Light-based bioprinting has not only benefits but also some drawbacks, e.g., biocompatible photopolymerizable materials are required, and printing conditions must be optimized for various cell types [117]. Researchers are actively working to address these challenges to broaden the applicability of light-based bioprinting in diverse tissue engineering contexts. Nevertheless, light-based bioprinting has promising potential for the future, as it is expected that additional developments in light projection technology, together with ongoing research into superior biomaterials and bioink formulations, will increase the accuracy and functionality of this method. Expanding the adaptability of light-based bioprinting might be facilitated by integration with sophisticated imaging modalities and the investigation of stimuli-responsive materials.

In the rapidly developing field of 3D bioprinting, light-based bioprinting is a noteworthy method that provides accurate, high-resolution production for tissue engineering applications. Light-based bioprinting has the potential to transform regenerative medicine by enabling the production of complex and biomimetic tissue constructs with unprecedented accuracy and efficiency, provided that researchers can overcome the aforementioned obstacles and explore new avenues.

### 2.5 Hybrid bioprinting

In order to capitalize on each technology's unique benefits, hybrid bioprinting combines many bioprinting techniques, including extrusion-based, inkjet-based, laser-assisted, and light-based bioprinting. This innovative combination aims to overcome the drawbacks of each individual bioprinting method to produce tissues with enhanced biomimicry, cellular vitality, and structural integrity [118]. By combining the strengths of each technique, researchers can harness precise extrusion capabilities, high-throughput droplet deposition, laser-induced precision, and light-based resolution in a synergistic manner. This integrative approach allows for the creation of complex, multi-material, and multi-cellular tissue structures [119].

The integration of several bioprinting methods has been optimized as a recent focus of improvements in hybrid bioprinting. Techniques include the creation of precise control systems to manage the simultaneous operation of many printing modalities and customized printing heads that can hold numerous bioinks [120,121]. These developments pave the way for the development of tissues with complex microarchitectures and improved physiological significance. The accuracy and dependability of hybrid bioprinting can be further improved by the integration of sophisticated imaging technologies and real-time monitoring systems [122]. In order to improve the process's flexibility and reproducibility, researchers are investigating the use of AI to dynamically modify printing settings in response to real-time input [123].

Hybrid bioprinting holds great potential, however, there are still issues in coordinating several printing modalities optimally and guaranteeing bioink compatibility. For this technology to advance, it is necessary that hybrid bioprinting techniques be standardized and that cross-contamination between various printing heads will be addressed. For this purpose, current investigations seek to improve integration tactics, broaden the range of bioinks that work well together, and investigate novel biomaterials [124]. Improvements in AI for adaptive control and the use of stimuli-responsive materials are expected to augment the accuracy and adaptability of hybrid bioprinting [125].

Hybrid bioprinting stands as the pinnacle of 3D bioprinting innovation, providing a distinct and synergistic approach to tissue engineering. Hybrid bioprinting is set to play a key part in the advancement of the field as researchers continue to address current obstacles and to investigate new possibilities in the field.

### 2.6 Mutual influence between bioprinting technologies and bioink formulations in BTE

Since bone tissue engineering is concerned with creating functional scaffolds, bioinks must meet several critical requirements related to printability, and also biocompatibility, mechanical properties, biodegradability, and surface characteristics [126]. One of the key properties is printability, as bioinks should have a certain viscosity and gelation kinetics to allow the formation of smooth extrusion and stable structures. Ideally, bioinks should be shear-thinning to allow easy flow through the nozzle, while maintaining their shape when printed, guaranteeing the formation of complex structures with precise geometry for bone scaffolding [127]. Moreover, the bioink formulation must be biocompatible to support the integration with the host tissue and it should support the adhesion and proliferation of osteogenic cells, facilitate the formation of ECM, and promote vascularization, which is critical in providing oxygen and nutrients to the regenerating tissue [128].

The mechanical properties of the printed structure are also important because the strength and stiffness of the scaffold should mimic those of natural bone [126]. This aspect can be modulated by the addition of reinforcing agents into the bioink, such as inorganic bone particles or calcium phosphates, for better mechanical characteristics [129,130]. However, it is also essential to consider that these properties change over time, since the scaffold should also be biodegradable. Ideally, the degradation of the scaffold is gradual and in parallel with new tissue formation, while the byproducts are harmless to the patient [126].

Additionally, cell behavior and scaffold functionality depend strongly on surface characteristics such as morphology, wettability, and porosity. Therefore, the ideal printed scaffold should provide interconnected porosity in the range of 200–350 μm to allow for the diffusion of oxygen, nutrients, and waste [131]. The surface morphology of the scaffold should be tailored to facilitate cell viability, proliferation, and differentiation in order to ensure tissue growth [132]. Finally, stimulus responsiveness is another aspect to be considered when fabricating bioinks for bone scaffolding, since bioinks with the ability of being responsive to external factors like pH, temperature, or mechanical stimulus, can support the regeneration process through changes in time [133]. Together, these properties guarantee that bioinks for BTE will enable cell growth, simulate the mechanical demands of bone, and degrade safely without interfering with tissue regeneration.

The bioprinting technologies and bioink formulations are deeply intertwined, as they affect the performance and outcome of each other. Each bioink formulation is tailored to different bioprinting methods, including extrusion-based, inkjet-based, laser-based, and light-based techniques, and thus, demands on the materials and functional properties of the bioink differ [134,135]. As a consequence, the feasibility, precision, and resolution of the bioprinting process depend on the composition and rheological characteristics of bioinks [136].

Specific mechanical and environmental stresses are introduced into bioinks with each bioprinting method. For instance, extrusion-based bioprinting, where the bioinks are forced through a nozzle, can impose significant shear stress on the cells within the ink, affecting cell viability and the structural fidelity of printed constructs. To protect cells and enable extrusion printing, bioinks require shear-thinning properties (i.e., viscosity decreases under shear stress) [137]. Furthermore, bioinks should undergo rapid post-printing crosslinking to convey mechanical stability to the printed structures [138].

Moreover, inkjet-based methods demand the use of low viscosity bioinks to produce consistent droplets [135]. Therefore, these bioinks must be balanced between fluidity, cell encapsulation efficiency, and structural integrity after deposition. Since cells are often subjected to thermal or mechanical stresses in droplet-based methods, bioinks are also required to shield cells from damage during the printing process. These formulations are often optimized for inkjet-based bioprinting, and include cell-supportive hydrogels that minimize exposure to these stressors [139].

As mentioned before, a highly precise method is laser-based bioprinting, where laser pulses eject the bioink onto a substrate. For applications involving small scale bone tissue constructs, high precision is required, and near single cell resolution is advantageous [134]. Nevertheless, to be suited for laser based printing, bioinks must be optimized for both viscosity and the capacity to absorb laser energy without harming the encapsulated cells. Bioinks have a critical influence on laser-based printing, as high energy laser pulses can produce heat that can harm cells or degrade sensitive bioink components [140]. Therefore, the materials used with this technology should be thermally stable for ideal outcomes.

Finally, stereolithography and related light-based techniques use photo-crosslinkable bioinks that solidify upon exposure to light. Therefore, these bioinks must be both photoresponsive and biocompatible to avoid damaging cells under light source or reactive photoinitiators [135]. In addition, bioinks employed in light-based bioprinting systems should have a relatively low viscosity for printability, transparency to ensure light penetration, and rapid gelation under light exposure [99].

Bioink formulations and bioprinting technologies are interdependent, with the demands of each method driving the design of tailored bioinks. At the same time, bioink properties influence the selection and optimization of bioprinting techniques for particular BTE applications.

## 3 Printable formulations for bone tissue engineering

In the field of tissue engineering and regenerative medicine, 3D bioprinting technology stands as a revolutionary technique aimed at transforming healthcare and personalized medicine. Central to the success of 3D bioprinting is the development and utilization of bioinks, which are highly important in creating functional tissue constructs by printing living cells and biomaterials layer-by-layer [141]. This chapter explores the use of commercial inks and lab-made (bio)inks, examining their compositions, properties, and the specific advantages they bring to the field of BTE. Through the exploration of case studies and the latest research, we aim to provide a comprehensive overview of the current landscape of printable formulations and their pivotal role in advancing regenerative medicine.

### 3.1 Commercial (bio)inks

In the rapidly evolving fields of tissue engineering and regenerative medicine, 3D bioprinting has distinguished itself as a transformative technology that has the potential to revolutionize the healthcare system. Therefore, the market for 3D bioprinting has witnessed substantial growth over the past decade due to an increasing gap between the demand for organ transplants and limited supply [142], as well as greater need for reliable in vitro models that can reduce dependency on animal use in toxicology testing [143]. The estimated size of the 3D bioprinting market was around $2.13 billion in 2022, and it is anticipated that this market will grow significantly up to $8.3 billion by 2030, with a compound annual growth rate of 18.51 % [144]. Rapid commercialization and growth of bioprinting technologies and associated products have highlighted its potential within this industry, thus creating new opportunities for both new entrants and established companies.

Some of the major manufacturers of 3D bioprinting and bioink market worldwide include: CELLINK, Advanced Biomatrix, Organovo Holdings Inc., Allevi Inc., REGENHU, TissueLabs, CollPlant Biotechnologies Ltd., Corning Inc., Merck, Jellagen, Humabiologics® Inc., 4D Medicine Limited, UPM Biomedicals, Inorigin Co., Ltd., Axolotl Biosciences, Akira Science, EnvisionTEC GmbH, Poietis, ROKIT Healthcare, Advanced Solutions Life Sciences, Regenovo Biotechnology Co., Ltd., Aspect Biosystems Ltd., Digilab Inc., GeSiM, Nano3D Biosciences, and Cyfuse Biomedical K.K.

The accuracy of 3D bioprinting largely depends on the development of bioinks, which are used to print cell-laden biomaterials, and on the optimization of bioinks for various bioprinting methods [145]. Although the field encompasses various formulations and techniques, commercial inks have gained popularity due to their standardized composition, reproducibility, and accessibility. Several commercial printable formulations are already available for 3D bioprinting, each being advertised as offering unique properties suited for different applications. Some commonly used commercial inks include.

- Gelatin methacryloyl: GelMA is produced from a denatured form of collagen and modified by methacryloyl groups, which permit photo-crosslinking, forming stable hydrogels upon ultraviolet (UV) light exposure in the presence of a photoinitiator [30,146]. Companies such as CELLINK and INNOREGEN, Inc. offer GelMA-based bioinks.
- Alginate: Alginate is a naturally occurring polysaccharide, usually extracted from brown algae in the form of sodium alginate (SA) [147], that can form hydrogels by ionic crosslinking with divalent cations, such as calcium ions [148]. Printable formulations based on alginate are highly biocompatible and capable of cell encapsulation. Commercial alginate inks are available from companies such as Advanced Biomatrix and REGENHU.
- Hyaluronic acid (HA): HA is also a polysaccharide and one of the main components of ECM, providing excellent biocompatibility and biodegradability [149,150]. HA-based bioinks are commonly used in areas where cell adhesion, migration, and proliferation are significant requirements [151]. Companies like Allevi and Merck offer commercial HA-based bioinks, such as HyStem and HyStem-C.
- Collagen: Collagen is a natural protein found in connective tissues and serves as a major structural component in the body [152,153]. Collagen-based bioinks offer biocompatibility and bioactivity, closely mimicking the native ECM [154,155]. Companies such as CollPlant and CELLINK provide collagen-based bioinks for 3D bioprinting applications.
- Fibrin: Fibrin is a fibrous protein involved in the blood clotting process [156]. Fibrin-based bioinks offer biocompatibility and promote cell adhesion and proliferation [157,158]. These bioinks are particularly suitable for applications requiring tissue regeneration and remodelling [159]. Companies such as Axolotl Biosciences offer commercial fibrin-based bioinks.
- Matrigel: Matrigel is a gelatinous protein mixture derived from Engelbreth-Holm-Swarm mouse sarcoma cells [160]. It contains various ECM proteins and growth factors, providing an environment conducive to cell growth and differentiation [161,162]. Although Matrigel is widely used in research, commercial options are available from companies such as Corning Inc. and Merck.

These are just a few examples of biomaterials found in commercially available inks used in 3D bioprinting. These formulations are not yet approved by the Food and Drug Administration, however, they represent an indication of the latest advancements in the field and address specific technical needs. Each formulation has its advantages and limitations, and the choice depends on the specific requirements of the tissue engineering application, including cell type, mechanical properties, and desired tissue functionality.

**Fig. 2:**
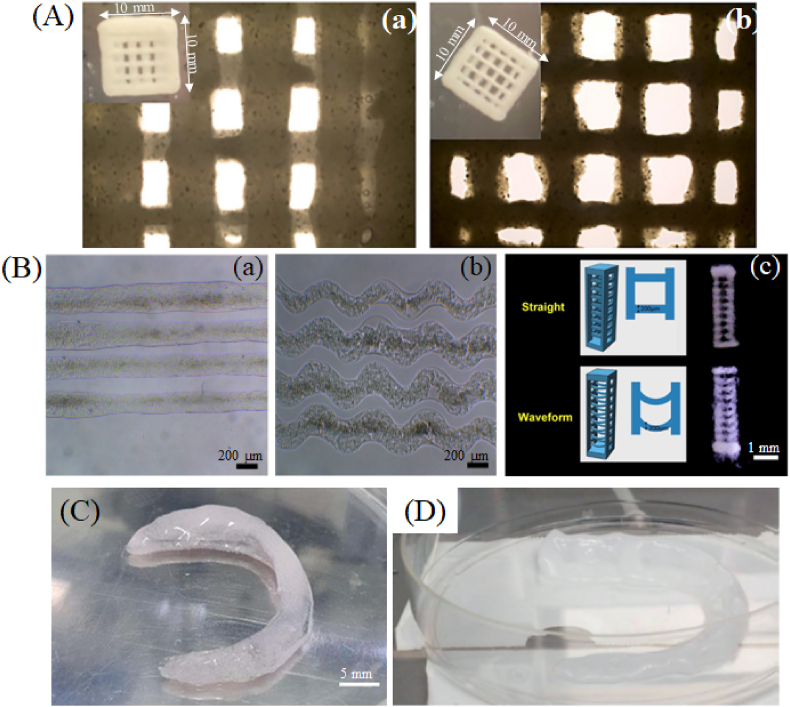
Scaffolds for bone tissue engineering 3D printed with commercial (bio)inks. (A) Optical images of 3D printed scaffolds using Ink-Bone from CELLINK (a) without cells and (b) with cells [163] (CC BY 4.0). (B) The microfibers obtained with Lifeink®200 as (a) straight and (b) waveform along with the (c) design models and 3D printed scaffolds [45] (CC BY 4.0). (C) Personalized meniscal scaffold 3D printed with Lifeink®200 reinforced with PCL/CNT nanofibers using the extrusion method [164] (CC-BY-NC). (D) Personalized meniscal scaffold designed with Lifeink®200 commercial ink, using the injet method [165] (CC BY 4.0).

So far, only a small number of commercial inks have been used in research for bone tissue engineering purposes (Table 1), mostly for cranio-maxillofacial regeneration and meniscus implants. A study by Glaeser et al. investigates the potential of commercial inks CELLINK BONE (Ink-bone) and GelXA BONE (GelXA) from CELLINK incorporating stromal cells to repair cranial bones [163]. The two inks have different compositions; namely Ink-bone contains nanofibrillated cellulose (CNF), SA, tricalcium phosphate, and proprietary buffer solution, while GelXA contains GelMA, SA, xanthan gum, lithium phenyl-2,4,6-trimethylbenzoylphosphinate (LAP), calcium phosphate tribasic, HAP, D-mannitol, and HEPES buffer solution, as described by the company. The objective of this study was to develop new techniques using extrusion-based bioprinting for the treatment of cranial bone loss and make use of cells that were derived from induced pluripotent stem cells (iPSCs). Both inks were combined with iPSC-derived neural crest cell‐mesenchymal progenitor cells (iNCC-MPCs) and bone marrow-derived mesenchymal stromal cells (BM-MSCs) respectively, at a ratio of nine parts ink to one part cell solution, and the resulting bioinks were used to fabricate scaffolds (Fig. 2 A) by extrusion, using the Bio X™ 3D printer from Cellink. A speed of 10 mm/s was employed when printing constructs designed as 10 × 10 × 2 mm squares at specific parameters, such as pressure between 25 and 30 KPa, concentration of 9 × 10^6^ cells/mL, and needle printheads made from plastic with a gauge (G) size varying between 22 G and 25 G. After printing, the scaffolds were crosslinked using a calcium solution from CELLINK, so that the bioinks could solidify. The in vitro investigations revealed that Ink-bone provided better results compared to GelXA in terms of cell viability and osteogenic differentiation of iNCC-MPCs and BM-MSCs. Particularly, bioluminescent imaging (BLI) signals increased from day 4 to day 28 in the case of Bone-ink, while for GelXA it was low on the first 7 days and increased after day 14. It was shown that alkaline phosphatase (ALP) levels were elevated in the group with BM-MSCs, osteocalcin (OCN) was expressed in higher levels in the group with Ink-bone and BM-MSCs, while osteonectin (ONN) gene expression was higher in the GelXA ink containing iNCC-MPCs. Moreover, in vivo experiments on NOD SCID mice showed that Ink-Bone combined with iNCC-MPCs and bone morphogenetic protein 6 (BMP-6) was the most successful formulation, increasing bone volume significantly, leading to tissue regeneration and defect repair after 8 weeks from surgery, demonstrating the potential value of such combinations for cranial reconstruction.

**Table 1:** Commercial printable formulations used in research for bone tissue regeneration.

Method	Producer/(Bio)ink	Cell line	Printing parameters	Crosslinking parameters	Application	Results	Ref.
Extrusion	Cellink/CELLINK BONE	BM-MSCs; iNCC-MPCs.	Gauge: 22; Pressure: 25–30 kPA; Temp.: NA; Speed: 10 mm/s.	Ca^2+^ (CaCl_2_ + D-mannitol + HEPES buffer solution) 30 s.	Cranial bone repair	High cell viability after 4 weeks; BLI signal increased from day 4 to day 28; Osteogenic differentiation in vitro and in vivo; Increased bone volume using iNCC-MPC-BMP-6-ink; Cell survival for 8 weeks after implantation;	[163]
	Cellink/GelXA BONE	BM-MSCs; iNCC-MPCs.	Gauge: 22; Pressure: 25–30 kPA; Temp.: NA; Speed: 10 mm/s;	Ca^2+^ (CaCl_2_ + D-mannitol + HEPES buffer solution) 30 s.	Cranial bone repair	High cell viability after 4 weeks; BLI signal increased from day 14 to day 28; Bone formation in vitro.	[163]
	Cellink/Cellink Bioink	hAD-MSCs	Gauge: 22-16; Pressure: 55–105 kPA without cells;Pressure: 75–85 kPA with cells;Temp.: NA.	100 mM CaCl_2_ 6 min	Cranio-maxillofacial regeneration	Best formulation: 77 % ink +23 % β-TCP; 80 % cell viability after 24h for the scaffold made with B23 as inner ink and Cellink + hAD-MSCs as outer ink;	[44]
	Advanced BioMatrix/Lifeink®200	PDL	Gauge: 34 G; Pressure: 250 kPa; Temp.: RT; Speed: 2 mm/s.	0.02 % riboflavin under UV light irradiation (365 nm), 30 min	Periodontal ligament regeneration	Cell attachment after 4h; Higher cell spreading and cell viability on waveform fibers; Upregulation of CCND1, CDH1, and POSTN.	[45]
	Advanced BioMatrix/Lifeink®200	MSCs	Nozzle inner diam.: 0.26 mm; Pressure: 25–30 kPA; Printhead temp.: 4 °C; Printing platform temp.: 37 °C Speed: 6 mm/s; Layer height:0.2 mm; Infill density: 40 %.	37 °C for 45–60 min.	Meniscus repair	Shrinking rate <2 %; Compressive modulus 18.7 ± 2.0 kPa; Cell viability >80 % after 7 days.	[164]
Inkjet	Advanced BioMatrix/Lifeink®200	BM-MSCs	Valve opening time: 190 μs; Dosing distance: 0.065 mm; Speed: 12 mm/s; Pressure: 20 kPa; Layer height: 0.25 mm; Infill density: 50 %.	No	Meniscus repair	50 % cell viability after 5 and 28 days; Cell proliferation after 28 days.	[165]

Another study by Walladbegi et al. focused on the 3D printing of scaffolds using commercially available inks for craniofacial bone defect repair [44]. Three compositions of ground beta tri-calcium phosphate (β-TCP) with CELLINK Bioink (CNF/Alg) were assessed in different combinations, namely Ink B23 (23 % β-TCP and 77 % Cellink), Ink B42 (42 % β-TCP and 58 % Cellink), and Ink B48 (48 % β-TCP and 52 % Cellink), with the last two being best suited for scaffold fabrication, as they can be printed easily with high stability. For these cell-laden constructs, human adipose tissue-derived mesenchymal stem cells (hAD-MSCs) were used at a concentration of 4 × 10^6^ cells/mL, and the bioprinting process involved a pneumatic-based extrusion system and a coaxial needle. The study examined three nozzle gauge configurations: 22–18, 22–16, and 22–14, indicating that 22–16 gauges were more effective at yielding reliable structures, and printing parameters for the scaffolds without cells included applying pressure of 105 kPa for the inner ink and 55 kPa for the outer ink, while the inks loaded with cells were printed at 75–85 kPa. Finally, the printed scaffolds were crosslinked by incubation in 100 mM calcium chloride solution for 6 min. The results show that the survival rate of cells within the printed constructs using Ink B23 as inner ink and CELLINK Bioink loaded with hAD-MSCs as outer ink was approximately 80 % after 24 h. Therefore, it was concluded that the application of a coaxial needle enabled the concomitant printing of ink and bioink into a stable scaffold, while maintaining the viability of cells and structural integrity. This could be an interesting approach for future consideration in the field of BTE, where there is a need for effective treatment methods that involve minimal invasion in craniofacial bone defects.

In the same area of cranio-maxillofacial applications, Lin et al. investigated the utility of Lifeink®200 (35 mg/mL type I collagen, pH 7) from Advanced Biomatrix in developing a biomimetic microfibrous scaffold for the regeneration of periodontal ligament (PDL) [45]. The authors fabricated 3D printed scaffolds in the form of straight and waveform microfibers (Fig. 2 B) using freeform reversible embedding of suspended hydrogels (FRESH) and in order to maintain the structure during the printing process, the scaffolds were obtained in a gelatin slurry support bath (GSSB). The extrusion method was chosen for this study, using the INKREDIBLE bioprinter from CELLINK, with optimized printing conditions: 250 kPa as pressure, 34 G as nozzle diameter, print speed of 2 mm/s, at room temperature (RT). The constructs were then crosslinked in 0.02 % riboflavin under UV for 30 min, followed by heating to 37 °C to remove the gelatin support. Immortalized human PDL cells were cultured at a seeding density of 4 × 10^3^ cells per microfiber and cultured in various shear stress conditions to investigate their behavior on printed scaffolds. It was shown that both collagen-based structures were obtained successfully using commercial ink and that waveform microfibers promoted higher cell viability, adhesion, and gene expression under shear stress (6 dyne/cm^2^) compared to straight microfibers. These results, especially the gene expression levels of cyclin D (CCND1), E-cadherin (CDH1), and periostin (POSTN), indicate that the developed waveform microfibrous scaffolds have great potential for PDL reconstruction, offering a biomimetic microenvironment that can withstand physiological loads and support tissue regeneration.

For meniscus repair, two studies investigated the use of Lifeink®200 by Advanced Biomatrix to fabricate 3D bioprinted meniscus-regenerative scaffolds. One study took place in 2022 when Stocco et al. explored the use of Lifeink®200 to develop a 3D bioprinted meniscus-regenerative scaffold [164]. In this case, the commercial ink was mixed with MSCs and used to fabricate scaffolds by extrusion bioprinting (BioEdPrinterV4, BioEdTech) either by itself or in combination with layers of nanofibers obtained from polycaprolactone (PCL) and carbon nanotubes (CNT) (hydrogel:nanofibers ratios of 2:1 and 4:1). The optimized printing parameters consisted of a nozzle inner diameter of 0.26 mm, printhead temperature of 4 °C, platform temperature of 37 °C, nozzle movement speed of 6 mm/s, layer height of 0.2 mm, and 40 % infill density. The fabricated scaffolds (Fig. 2 C) were kept at 37 °C for 45–60 min so that crosslinking of collagen hydrogel to take place. According to the authors, adding aligned PCL/CNT nanofibrous mats between the hydrogel layers in a ratio of 2:1 led to the best results, improving the mechanical properties and structural integrity of the scaffold without affecting cell viability. The structural robustness of the scaffolds fabricated with nanofibers was demonstrated by a shrinking rate (post-crosslinking) below 2 %. Mechanical tests were also performed and showed that the compressive modulus was significantly higher in scaffolds with nanofiber layers (18.7 ± 2.0 kPa) than in those without them (3.9 ± 2.2 kPa), while cell viability assays (live/dead) showed that over 80 % of MSCs remained viable after being cultured for 7 days for all samples.

Another study was conducted by Filardo et al. in 2019 with the aim of developing a patient-tailored, cellularized human meniscus through 3D bioprinting [165]. The bioink was obtained by loading the collagen ink with BM-MSCs (38 × 10^6^) in a 3 mL syringe, and the 3D bioprinting was performed using the 3D Discovery printing equipment produced by REGENHU with a microvalve-based inkjet printhead. The authors used magnetic resonance imaging data from a healthy volunteer for the medial meniscus, that was processed to create a 3D model, and the meniscus was printed in a rectilinear filling pattern with 50 % infill density in a layer-by-layer method. The printing parameters were set as follows: valve opening time set at 190 μs, 0.065 mm dosing distance, 12 mm/s speed, 20 kPa pressure, and 0.25 mm layer height. To provide optimal conditions for cell viability and to avoid the crosslinking of collagen, the cartridge was kept at room temperature, and the bioprinting was performed in a culture medium heated to 37 °C. The results of this study indicate good printability and shape fidelity for the bioprinted structure (Fig. 2 D), as well as a cell viability of 50 % after 5 days. The authors report that the remaining cells were alive after 28 days (according to live/dead assay) and that they were able to proliferate and migrate in the artificial meniscus. Even though the mechanical properties were not investigated, this work demonstrates that the commercial formulation is reliable for this type of application and that it is feasible to manufacture personalized cell-laden scaffolds for bone tissue regeneration. Both studies show that Lifeink®200 offers great promise for creating patient-specific meniscus scaffolds that closely resemble native tissues in terms of their structure and function, either by extrusion or by inkjet bioprinting technology.

The diverse range of commercial inks available, based on GelMA, SA, HAP, collagen, fibrin, etc., offer unique properties for various applications in tissue engineering. In particular, studies have demonstrated the effectiveness of these commercial inks in BTE applications, i.e., cranio-facial bone defect repair, periodontal ligament regeneration, and meniscus repair. For instance, research involving CELLINK BONE and GelXA BONE inks has shown promising results in cranial bone regeneration, while the use of Lifeink®200 has been effective in fabricating meniscus-regenerative scaffolds. These findings highlight the potential of commercial inks in advancing 3D bioprinting applications, offering significant benefits in terms of biocompatibility, cell viability, and tissue regeneration, and paving the way for innovative and patient-specific therapeutic solutions.

### 3.2 Lab-made (bio)inks

Lab-made inks and bioinks for 3D printing in BTE represent a significant advancement in regenerative medicine by enabling the creation of customized bone grafts. These printable formulations, which often combine synthetic polymers, ceramics, and natural polymers such as agarose (Aga), SA, gelatin, collagen, and chitosan, are designed to replicate the complex structure and functionality of natural bone [166]. The incorporation of living cells in printable mixtures further enhances their potential by promoting cell growth and tissue regeneration [126]. However, the road to commercialization presents several challenges, i.e., regulatory approval requires rigorous testing to ensure safety and efficacy, and there is a need for extensive clinical trials to demonstrate long-term benefits and viability [26,167]. Despite these obstacles, ongoing research, collaboration between academia and industry, and advancements in 3D printing technology are paving the way for these innovative solutions to reach the market, offering alternatives for effective and personalized bone repair treatments. In this chapter, several printable mixtures based on agarose, SA, gelatin, collagen, and chitosan are presented and discussed in terms of printing method and parameters, results, and application (Table 2).

**Table 2:** Lab-made printable formulations with applications in BTE.

Method	Formulation	Cell line	Printing parameters	Crosslinking parameters	Application	Results	Limitations	Ref.
Extrusion	MAga	MSC	Nozzle: 600 μm inner diam.; Pressure: NA; Nozzle temp.: 35 °C; Bed temp.: 26 °C; Speed: 2 mL/min.	UV light for 5 min.	Hard tissue engineering	Good printability; Cell viability after 5 days; Cell proliferation; Cell attachment; Osteogenic differentiation.	No in vivo testing; Compressive strength low compared to anatomical bone.	[168]
	Aga/HAP/GO	hMSC	Gauge: 23; Pressure: NA; Temp.: RT; Speed: 0.2 mm/s.	NA	Large bone defects reconstruction	Good biocompatibility; Cell viability 88 %; Cell adhesion and proliferation; Osteogenic markers expression.	No in vivo testing; No studies on mechanical properties.	[46]
	PCL/Aga	L-929	Gauge: 27; Pressure: NA; Temp.: heated nozzle; Speed: 7 mm/s.	NA	Tissue engineering	Compressive modulus: 0.44 ± 0.2 mPa; Cell adhesion; Good cell viability;	No in vivo testing; Aga decreases mechanical properties.	[172]
	Aga/SA/BG	MC3T3-E1	Gauge: 21 G; Pressure: NA; Temp.: RT; Speed: 30 rpm.	Chelation-based	Bone repair	Cell growth; Cell proliferation;	No in vivo testing; Zn and Cu can cause cell harm and toxicity.	[173]
	SA/PVA/hACM	C20A4	Gauge: 22; Pressure: NA; Temp.: NA Speed: NA; Infill density: 50 %.	500 mM CaCl_2_, 30 min	Cartilage repair	Good printability; Cell viability: 87 % (day 1), 76 % (day 7), 86 % (day 14); Expression of COL2A1, SOX9.	No in vivo testing; No long-term testing of stability and functionality; Possible immune response.	[174]
	CNC/SA/Gel	hBMSCs	Gauge: 27; Pressure: 200–500 kPA; Printhead temp.: 35 °C; Printing platform temp.: 4 °C Speed: 5 mm/s;	100 mM CaCl_2_, 60 min	Bone healing	Good printability; Cell adhesion; Cell proliferation into the scaffold; Cell viability >70 % (seeding); Osteogenic markers expression; Osteogenesis after 3 weeks.	Low swelling potential; No mechanical studies performed on the printed scaffolds.	[43]
	SA/Gel/β-TCP	MG-63	Gauge: 22; Pressure: 60–200 kPA; Temp.: NA; Speed: 2 mm/s.	100 mM CaCl_2_, 10 min	Bone regeneration	Printing accuracy; 5 % degradation; Increased cell viability; ALP activity: 27 nmol/well.	No in vivo testing; Possible inflammatory reaction.	[130]
	SA/CMC/58S BG	SaOS-2	Gauge: 22; Pressure: 30 kPA; Temp.: 37 °C; Speed: 2 mm/s;	4 % CaCl_2_ 0.5–5% FeCl_3_, 15 min	Bone regeneration	Young's modulus: 173 kPa; Pore printability: 67–99 %; Cell viability and proliferation highest with 1 % Fe^3+^; ALP activity higher on 0.5 and 1 % Fe^3+^.	No in vivo testing; Low Young modulus compared to anatomical bone.	[175]
	PRF/NP/SA	rBMSCs MC3T3-E1	Nozzle inner diam.: 0.41 mm; Pressure: 1.4–1.8 bar; Temp.: NA; Speed: 2 mm/s.	100 mM CaCl_2_ after every layer and 10 min at the end	Bone regeneration	Shear stress ≥300 Pa; PRF increased cell adhesion; Cell viability over 80 %; ALP activity: 250 units/L.	Increasing the NP/SA ratio decreases compressive strength and compressive modulus; Increasing the NP/SA ratio increases degradation rate.	[176]
	Alg/Ma-dECM	hASCs	Nozzle inner diam.: 0.25 mm; Pressure: 100 kPa; Temp.: NA; Speed: 10 mm/s.	10 % CaCl_2_ (aerosol spraying) + UV light + immersion in 2 % 10 % CaCl_2_	Bone tissue engineering	Cell viability over 90 %; Higher cell proliferation and osteogenic activity than pure Alg.	No in vivo testing; Higher dECM concentration increases viscosity and decreases cell viability.	[177]
	ADA-Gel/FS	MC3T3-E1	Nozzle inner diam.: 0.4 mm; Pressure: 100–190 kPa; Temp.: 30 °C; Speed: 10 mm/s.	10 % w/v mTG in 0.1 M CaCl_2_, 15 min, 22 °C.	Bone tissue engineering	Good shape fidelity and printability; Young's modulus: 96 ± 8 kPa; Swelling: 350 % in 3 days; Stability: 35 days. Apatite formation in 28 days; Cell viability over 90 %;Increased ALP activity for 28 days	No in vivo testing; Structural integrity could be affected by excessive swelling.	[178]
	SA/Chi/Gel	SaOS-2	Nozzle diam.: 0.8 mm; Speed: 10 mm/min; Layer height: 0.6 mm; Infill density: 40 %.	1.36 mol/L CaCl_2_, 1h; 3 % EDC-NHS, 24 h, 4 °C.	Bone tissue engineering	Swelling decreased with Gel content; Degradation increased with Gel content; Apatite formation in 7 days; Tensile strength: 693 ± 15 kPa; Protein adsorption; Cell proliferation and attachment – day 7; Scaffold biomineralization.	No in vivo testing; Tensile strength low compared to anatomical bone.	[179]
	Gel/CMC/Alg	MG-63	Nozzle inner diam.: 0.41 mm; Pressure: 25–55 kPA; Temp.: 37 °C; Speed: 10 mm/s;	6 % CaCl_2_	Meniscus repair	Good mechanical stability and printability; Swelling higher in PBS than SBF; Degradation: lower rate in PBS than SBF; Apatite formation; UCS: 0.165 MPa; Compressive modulus: 2.929 MPa; Cellular proliferation increased with time;	No in vivo testing; Need for in-situ simultaneous crosslinking for direct 3D bioprinting.	[180]
	Gel/HAP/gly	hASCs	Nozzle inner diam.: 0.25 mm; Pressure: 90–110 kPa; Temp.: 33–34 °C; Printing platform temp.: 4 °C Speed: 10 mm/s.	100 mM EDC/NHS, 24 h, 4 °C.	Hard tissue regeneration	Printability improved with gly; Higher cell proliferation and distribution with gly; Expression of COL-I and RUNX2.	No in vivo testing; Compressive modulus is low compared to human bone.	[181]
	GelMA/Gel/HAP	MC3T3-E1	Gauge: 22; Pressure: NA; Temp.: 30 °C; Speed: NA; Crosshatch infill: 50 %.	UV light for 10 s	Bone defect repair	Swelling decreased with HAP content; Degradation decreased with HAP content; Increased cell proliferation; Cell viability over 76 %; ALP activity increased by day 28; BMP-7 and OCN expression.	No in vivo testing; Swelling behaviour is not tunable; Degradation in less than 28 days.	[182]
	PCL/β-TCP/nHA/MgO/Gel-RSV	hUC-MSCs	Gauge: NA; Pressure: NA; Temp.: 130 °C; Speed: 1.5 mm/s.	10 % v/v GA vapor, 16 h	Bone regeneration	Compressive strength: 25 MPa and 20 MPa after degradation; Elastic modulus: 48 MPa and 42 MPa after degradation; Increased cell viability; Increased ALP activity; Bone healing in vivo: 86 %.	Gel accelerates clot formation; Gel and RSV decrease mechanical properties.	[183]
Inkjet	GelMA/PeMA/CNF/GO	NCTC L-929	Valve opening time: 800/1000 μs; Valve closing time: 1800/2500 μs; Dosing distance: 0.1 μs; Feed rate: 10 mm/s; Pressure: 30–35 kPa; Printing delay: 100 ms; Infill density: 15 %.	UV light for 5 s	Bone regeneration	Viscosity increases with GO concentration; Lattice fidelity: 82 %; Height fidelity: 99 %; Degradation: residual GF 76 %; Cell viability increased with GO concentration;	No in vivo testing; High GO content affects printability.	[184]
Extrusion	MA/BC/Gel	MC3T3-E1	Nozzle: 0.5 mm; Pressure: NA; Temp.: RT; Speed: 1.5 mm/s.	EDC/NHS (15 mmol/L/6 mmol/L), 12 h.	Bone tissue engineering	Good printability; Young's modulus: 11.48 GPa; Swelling ratio: 338 %–524 %; Compression modulus: 0.716 MPa; Enhanced cell viability; Higher ALP activity; Higher levels of COL-I and RUNX2.	No in vivo testing; Mechanical properties lower compared to anatomical bone.	[186]
	Gel/DB	MC3T3-E1	Nozzle: 0.4 mm; Pressure: 120–180 kPa; Temp.: 25 °C; Speed: 5 mm/s.	10 % w/v mTG, 15 min, R.T, and overnight at 4 °C.	Bone tissue engineering	Good printability with 1 % Gel and 3 % DB; Young's modulus: 27 ± 3 kPa - 29 ± 3 kPa; Gel/1 % DB - fastest relaxation; Swelling ratio: 70 % for Gel/5 % DB; Highest degradation rate: Gel/5 % DB; Cell viability: highest on Gel/1 % DB – day 21; Cell proliferation: lowest on Gel/5 % DB.	No in vivo testing; A higher DB content affects printability; Mechanical properties lower compared to anatomical bone.	[187]
	Gel/DB	MC3T3-E1 hMSCs	Nozzle: 0.41 mm; Pressure: 150–180 kPa; Temp.: 25 °C; Speed: 10 mm/s.	10 % w/v mTG, 15 min, RT.	Bone tissue engineering	Viscosity: 170.67 Pa s (increased with DB content); Optimal printability: Gel/5 % DB (45 μm); DB increased cell viability; Highest cell proliferation: Gel/10 % DB (45 μm); Highest ALP acitivity: Gel/10 % DB (45 μm);	No in vivo testing; A higher DB content affects printability; A lower DB content decreases cell proliferation.	[129]
	Liquid Col.	L-929	Gauge: 27; Pressure: 10 kPa; Printhead temp.: 4 °C; Speed: 3 mm/s.	EDC/NHS or genipin	Bone regeneration	Thermal stability: 71 °C (10 mM EDC-NHS) and 73 °C (10 mM genipin); AuNPs conjugation: 100 % with EDC-NHS and 78 % with genipin; Cell viability highest for Col-genipin-AuNPs;	No in vivo testing; Slight decrease in biocompatibility after crosslinking with EDC/NHS.	[42]
	CCL	hOP	Nozzle: 0.84 μm; Pressure: NA; Temp.: NA; Speed: 2 mm/s. Layer overlap: 0.66 μm; Extrusion rate: 1.1 μL/s;	EDC	Bone tissue engineering	Yield stress increased with Col. Concentration; Denaturation temperature: 114.72 °C; Maximum tensile stress: 2.65 ± 1.89 MPa; Increased cell viability; Higher ALP expression;	No in vivo testing; Maximum tensile stress lower than control.	[188]
	Col/HAP	BMSCs	Gauge: 22; Pressure: NA; Bath temp.: 4 °C; Speed: 1 mm/s.	NA	Bone tissue engineering	Compressive modulus before lyophilization: 0.39 ± 0.11 MPa; Compressive modulus after lyophilization: 25.2 ± 3.1 MPa; High elasticity (nearly 100 % recovery); Cell viability increased 3 times after 7 days; Higher ALP expression; Cell adhesion and distribution.	No in vivo testing; Low printability without support bath; Low mechanical properties compared to anatomical bone.	[189]
	Col/β-TCP	hASCs/HUVECs	Nozzle inner diam: 250 μm; Pressure: 120 ± 10 kPa; Barrel temp.: 20 °C; Plate temp.: 37 °C; Speed: 10 mm/s.	Genipin, 37 °C, 1 h.	Spinal fusion; Bone tissue regeneration.	Compressive modulus: 9.6 ± 3.1 kPa; Cell viability: 93 % after 1 day; Higher cell proliferation; Higher expression of osteogenic biomarkers; In vivo bone formation (fusion rate 89.9 %); Blood vessel formation (27.8 % immunoreactivity).	Low compressive modulus compared to trabecular bone; Addition of β-TCP requires a higher extrusion pressure which affects cell viability.	[190]
	Col/GelMA	NA	Gauge: 25, 27; Pressure: 6–22.3 PSI; Temp.: 20 °C; Speed: 4–6 mm/s.	UV light, 3 min.	Cartilage tissue engineering	Optimum formulation: GelMA with 1 % Col; Swelling ratio: 400–500 %;	No in vivo testing; A higher Col content (2 %) affects printability and crosslinking process.	[191]
	Col/Chi	BMSCs	Nozzle: 0.26 mm; Pressure: 0.2–0.48 MPa; Printhead temp.: 8 °C; Platform temp: 10 °C; Speed: 13–18 mm/s.	3 mM genipin, 37 °C, 1.5 h.	Bone tissue engineering	Young's modulus: 61.980 ± 10.139 kPa; Degradation: 30 % (2 h) and 50 % (24 h); Cell viability: over 97 %;	No in vivo testing; Low mechanical properties compared to anatomical bone.	[47]
	Col/THA/CaP	L-929; hMSCs	Gauge: 22; Pressure: 0.2–0.8 bar; Temp.: NA; Speed: 4 mm/s.	UV light	Bone regeneration	Swelling ratio: 150 %; Degradation decreased with CaP content; Compressive modulus: 290–446 kPa; High cell viability; Osteogenic differentiation.	No in vivo testing; Low compressive modulus; Relatively fast degradation.	[192]
	ColMA/tHA/PEGDA	NC	Gauge: 22; Pressure: NA; Temp.: NA; Speed: NA; Infill rate: 30 %.	UV light, 90 s.	Cartilage tissue engineering	Good printability; Cell viability post-printing: 85.71 % ± 0.6 %; Cell viability post-UV: 83.3 % ± 1.2 %; Swelling ratio: 27.7 %; Chondrogenic gene expression.	No in vivo testing; Addition of tHA and PEGDA decreased the compressive modulus.	[193]
	Chi/nHAP	MC3T3-E1	Gauge: 27; Pressure: 50–80 kPa; Printhead temp.: RT; Printbed temp.: 35 °C; Speed: 4–12 mm/s;	GP and SHC	Osteochondral tissue regeneration	High printability; Compressive modulus: 45.6 ± 4.9 kPa (40 % nHAP); Cell viability post-printing: over 90 %; Degradation: slowest woth 40 % nHAP.	No in vivo testing; Chi gelation is time dependent affecting multi-layer bioprinting; | No evaluation of degradation in time and of mechanical properties.	[195]
	Chi/CNC	MC3T3-E1	Gauge: 20; Pressure: 10–30 kPa; Printhead temp.:25 °C; Printbed temp.: 37 °C; Speed: 0.5–2 mm/s;	Hydroxyethyl cellulose	Meniscus repair	Yield stress: 400–585 Pa; Cell viability: over 90 %; ALP activity: day 7; ECM formation; Ca mineralization: day 21.	No in vivo testing; Addition of cells in the bioink decreased the recovery of storage modulus.	[196]
	Chi/βGP/Gel-SNA/nHAP	MG-63 L-929	NA	5.6 % βGP and 0.1 M NaHCO_3_	Bone tissue engineering	Elastic modulus: 15.5 kPa; Water uptake: 2.5 %; Degradation rate: 3 weeks; Cell viability: over 88 %; ALP activity: 36.8 units/mL; Ca deposition: 28.76 ± 1.78 μg/mg	No in vivo testing; Addition of nHAP leads to a decrease in pore size; A higher nHAP content might affect the complete gelation between Chi and βGP.	[197]
	Me/gly/Chi	MG-63	Gauge: 26; Pressure: 120 ± 10 kPa; Temp.: RT; Speed: 6 mm/s.	12 μM riboflavin, visible light 30 s.	Bone tissue engineering	Compressive modulus: 25.7 kPa; Cell viability: 92 %; Low swelling ratio; Highest ALP activity; Ca deposition in 7 days.	No in vivo testing; Low mechanical properties compared to anatomical bone; Curing for more or less than 70 s leads to poor printability.	[198]

#### 3.2.1 Agarose-based (bio)inks

Agarose-based inks have shown significant promise for bone tissue engineering due to their unique properties, such as biocompatibility, ease of gelation, and support for cellular activities. Several recent studies have investigated these printable formulations and the combination of agarose with other materials, such as PCL, bioglass (BG), metal ions, graphene oxide (GO), and HAP, in order to fabricate scaffolds suitable for hard tissue engineering. Mukundan et al. [168] explored the use of human placenta-derived MSCs in agarose-based bioinks. Several bioink formulations were investigated and the ideal one included agarose methacrylate (MAga) with a 24 % degree of substitution and 5 % w/v hydrogel concentration mixed with MSCs (3.6 × 10^6^ cells/mL), exploring the potential of placenta-derived MSCs to differentiate into osteoblasts [169]. The bioprinting process was achieved using an extrusion-based method to obtain cell-laden 3D scaffolds and the results of the study show that the bioink led to obtaining scaffolds with good printability that supported cell viability, proliferation, and attachment after 5 days, as well as cell differentiation after 14 days, with enhanced expression of osteopontin (OPN) and expression of OCN. This approach represents a new way of involving human-derived cells to create a biologically active environment suitable for bone tissue regeneration.

Yadav et al. [46] investigated agarose-based inks reinforced with GO and HAP for bone regeneration. This printable formulation aimed to combine the osteoconductive properties of HAP [170] with the mechanical reinforcement provided by GO [171]. In the study, an extrusion-based 3D printer was employed to fabricate the scaffolds, and the results indicated that the presence of GO significantly enhanced the mechanical strength and osteogenic potential of the ink. The scaffolds showed high cell viability (over 87 %), proliferation, and increased expression of osteogenic markers such as BMP, Runt-related transcription factor 2 (RUNX2), COL-I, OPN, and OCN. This combination of materials provided a promising strategy for developing personalized bone grafts. Another group focused on combining agarose with a synthetic polymer, namely PCL, for the fabrication of composite scaffolds [172]. The 3D printing process used was extrusion-based, which allowed the integration of the mechanical strength provided by PCL with the biocompatibility provided by agarose. The results showed that the composite scaffolds had good mechanical properties and supported cell viability and adhesion. The incorporation of PCL improved the structural integrity of the scaffolds, making them more suitable for load-bearing applications in BTE.

Another approach was recently presented by Bhattacharyya et al. [173], reporting a printable formulation developed by combining agarose with SA, bioglass nanoparticles, and essential trace elements (iron, zinc, and copper) for BTE, using an advanced twin-screw extrusion-based 3D bioprinter to create 3D scaffolds. The incorporation of bioglass nanoparticles improved the mechanical properties, such as compressive strength, and enhanced the biocompatibility and osteogenic potential of the scaffolds. It was shown that the addition of metal ions further supported cell proliferation and differentiation. The results indicated significant improvements in cell growth and ECM production, making the proposed ink suitable for bone regeneration applications.

#### 3.2.2 Alginate-based (bio)inks

**Fig. 3:**
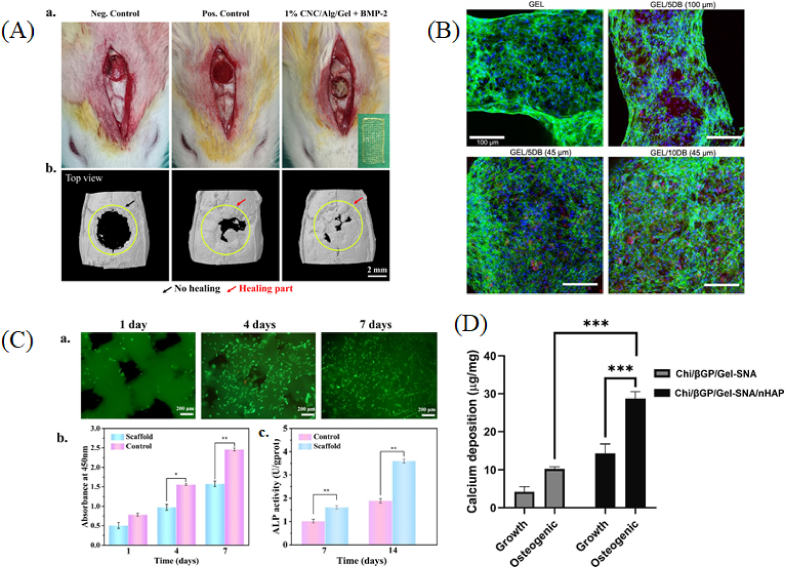
Lab-made printable formulations for BTE. (A) Images of in vivo scaffold implantation (a) and μCT images showing the results in rat calvaria defect model for bone regeneration after 3 weeks from transplantation (b). Black arrow - no healing and red arrow - healing part. Reprinted from Ref. [43] with permission from Elsevier. (B) Confocal microscopy images showing the growth of hMSCs inside the 3D bioprinted Gel and Gel/DB scaffolds after 28 days. Scale bars: 100 μm [129] (CC BY 4.0). (C) Characterization of biocompatibility and osteogenic properties of scaffolds: (a) fluorescence miscroscopy images of BMSCs, scale bars: 200 μm; (b) the optical density value of cells cultured on the control and Col/HAP scaffolds for 1, 4 and 7 days; (c) ALP activity after BMSCs were cultured on the control and Col/HAP scaffolds for 7 and 14 days [189] (CC BY 4.0). (D) Calcium deposition in bioprinted scaffolds obtained with Chi/βGP/Gel-SNA and Chi/βGP/Gel-SNA/nHAP bioinks cultured with MG63 cells for 14 days in growth media and osteogenic media. Reprinted from Ref. [197] with permission from Elsevier. (For interpretation of the references to colour in this figure legend, the reader is referred to the Web version of this article.)

Alginate-based bioinks have been widely investigated for BTE applications due to their biocompatibility, biodegradability, and ease of gelation. Recently, Aitchison et al. [174] developed a bioink composed of SA, polyvinyl alcohol (PVA), human articular cartilage matrix (hACM), and C20A4 cells (5 × 10^6^ cells/mL) to create scaffolds for BTE. The study aimed to utilize the biological components of hACM to enhance cell proliferation and differentiation, and the bioprinting method employed an extrusion-based approach to fabricate the scaffolds. The results showed that the inclusion of hACM significantly improved the biological properties of the bioink, leading to enhanced cell viability, proliferation, and chondrogenic differentiation, as demonstrated by the expression of chondrogenic genes collagen type 2 alpha 1 (COL2A1), sex-determining region Y-box 9 (SOX9), and glyceraldehyde-3-phosphate dehydrogenase (GAPDH). This bioink demonstrated potential for applications where the regeneration of cartilage tissue with improved biological functionality is required. Moreover, Dutta et al. [43] explored a composite ink made from SA, gelatin, and cellulose nanocrystals (CNC), where the authors aimed to capitalize on the mechanical and biological benefits provided by CNC. Using an extrusion bioprinting technique, the study showed that the formulation not only demonstrated good printability and structural integrity but also supported cell viability (over 70 %), growth and differentiation (expression of RUNX2, BMP-2, ALP, OCN, OPN, bone sialoprotein, osterix (OSX), and COL-I), providing a favorable environment for bone tissue regeneration. The scaffolds obtained based on the aforementioned composite ink were also investigated in vivo by implantation in ICR male rats model with a 5 mm critical defect in the calvaria bone and the results demonstrated good biocompatibility of the scaffolds, no signs of inflammation, and bone formation occurred within a span of 3 weeks (Fig. 3 A).

With the aim of enhancing the physical properties of a scaffold, of promoting cell viability, and facilitating bone regeneration, Wu et al. [130] developed a printable formulation consisting of 10 % SA, gelatin, and β-TCP for 3D printing. The authors reported that the printed scaffolds exhibited excellent cell viability, ALP activity (27 nmol/well), and osteoblast differentiation, demonstrating their potential for personalized bone regeneration treatments. The SA /Gel/β-TCP scaffold also showed promising results in terms of mechanical support and biodegradability, making it suitable for clinical applications in dental bone regeneration.

Moreover, Tahmasebifar et al. [175] explored a bioink composed of SA, carboxymethyl cellulose (CMC), 58S bioglass, and SaOS-2 cells (3 × 10^6^ cells/mL). The dual crosslinking with calcium and iron ions aimed to improve the mechanical properties of the hydrogel. The study found that the crosslinking density and resultant rigidity achieved with optimal Fe^3+^ concentrations significantly enhanced cell viability and osteogenesis. The bioprinted scaffolds demonstrated superior mechanical properties and biological activity, demonstrating their efficacy for bone regeneration applications. More recently, Liu et al. [176] proposed a printable formulation consisting of SA and nacre powder (NP) to create scaffolds via 3D printing. To enhance bone regeneration, platelet-rich fibrin (PRF) was loaded onto the scaffolds, and the structures exhibited suitable porosity, degradation performance, and mechanical properties, mimicking bone tissue. In vitro results showed good cell viability, proliferation, and adhesion after 7 days, and ALP activity measured at 250 units/L, while in vivo experiments on New Zealand rabbits demonstrated the healing potential of this printable formulation for skull bone regenration. Specifically, scaffold implantation did not cause an inflammatory response and it started to show signs of bone regeneration after 4 weeks, initially at the marginal area of the bone defect. At 8 weeks post-implantation, a significantly higher new bone volume ratio was observed, along with collagen deposition and high expression of COL-I, vascular endothelial growth factor (VEGF), and RUNX2 signaling molecules. Finally, trabecular bone structures were observed and the results indicated that the inclusion of PRF further accelerated bone regeneration, making this composite ink a promising candidate for clinical applications in bone tissue repair.

Particularly in the field of BTE, bone-derived dECM can serve as a scaffold that retains the key components of the native tissue ECM, including COL-I and glycosaminoglycans, which are essential for osteogenesis, and enhance osteogenic differentiation, especially in combination with other materials such as alginate. In a study by Lee et al. [177], it was demonstrated that this formulation based on sodium alginate and dECM improved printability, mechanical strength, and bioactivity over conventional hydrogels, allowing for the fabrication of 3D cell-laden structures that support bone regeneration. To be precise, the bioink, composed of methacrylated dECM (Ma-dECM), sodium alginate, and human adipose derived stem cells (hASCs) in concentration of 5 × 10^6^ cells/mL, was optimized for enhanced cell viability and osteogenic differentiation by adjusting the Ma-dECM concentration (0.05 g–0.4 g) and crosslinking conditions. The formulation containing 0.2 g Alg/Ma-dECM provided the best results, i.e., over 90 % cell viability and significantly enhanced osteogenic markers like ALP, BMP-2, OCN, and OPN, making it a highly effective platform for BTE applications.

#### 3.2.3 Gelatin-based (bio)inks

Recent advancements in bone tissue engineering have explored the potential of gelatin-based bioinks, combining the natural properties of gelatin with various additives to enhance mechanical strength, biocompatibility, and osteoinductivity.

For bioprinting applications, several studies have explored the combination of gelatin and alginate, among other components, to improve the mechanical properties of gelatin. The study by Özenler et al. [178] focused on a bioink composed of alginate dialdehyde (ADA) and gelatin, with the addition of fish scale (FS) particles (1 %, 3 %, 5 %, 10 %) and MC3T3-E1 cells (5 × 10^6^ cells/mL). This bioink was designed to mimic the natural ECM and enhance the osteogenic differentiation of stem cells, and it was prepared using a crosslinking process involving the oxidation of alginate, which improved the mechanical properties and printability of the bioink. The bioprinting was performed using an extrusion-based method, which allowed for the creation of complex scaffold structures. Results from in vitro studies demonstrated that the fabricated bioink supported cell viability (over 90 % after 14 days) and proliferation, and the mechanical properties improved with the concentration of FS particles, at 10 % measuring the highest Young's modulus (96 ± 8 kPa), the highest relaxation time, and the highest swelling capacity (350 %). Moreover, the bioink further promoted osteogenic differentiation and mineralization, as shown by the formation of apatite on the surface of the scaffold and by an increase in ALP activity and OCN expression. In another study by Singh et al. [179], a tri-polymer complex ink composed of SA, chitosan, and gelatin (SA/Chi/Gel) was developed. The scaffolds were fabricated using extrusion-based 3D printing, resulting in porous structures with excellent mechanical stability. The authors reported that increasing gelatin content (0–15 %) led to an improvement in tensile strength (from 386 ± 15 kPa to 693 ± 15 kPa), increased degradation, and decreased swelling ratio. Moreover, it was found that higher gelatin content significantly increased protein adsorption, cell metabolic activity, cell attachment, cell density, and cell proliferation, as well as biomineralization. Moreover, Sathish et al. [180] investigated a bioink composed of gelatin, CMC, and alginate for direct and indirect 3D printing applications. This tricomposite bioink was obtained by mixing the formulation with MG63 cells (1 × 10^5^ cells/mL) and was optimized for printability and mechanical properties (35 % Gel, 2 % CMC, 4 % Alg) to create scaffolds for cartilage tissue engineering, specifically for human meniscus repair. The addition of CMC provided additional structural support and improved the biomechanical properties of the scaffolds. The study utilized both direct extrusion-based bioprinting and a negative mold approach to fabricate the scaffolds, demonstrating that the tricomposite bioink could be used effectively in both methods. The authors reported good mechanical properties for the bioprinted scaffolds, with an ultimate compressive strength (UCS) of 0.165 MPa and a compressive modulus of almost 3 MPa, as well as favorable biological properties, the scaffolds showing good cell viability, proliferation, and ECM secretion. These studies highlight the potential of gelatin-based bioinks with alginate for BTE, demonstrating improved mechanical properties, printability, and biological performance through various compositional adjustments and bioprinting techniques.

In search of a printable formulation that mimics the natural bone matrix and supports osteogenic processes, scientists combined gelatin with HAP. Kim et al. [181] explored a formulation composed of these two components and found that by itself, the ink had poor printability because of HAP sedimentation and nozzle clogging. However, the addition of 30 vol% glycerol (gly) increased the viscosity of gelatin and the temperature of sol-gel transition, resulting in significantly improved composite ink printability and stability. The printing method employed was extrusion-based, enabling the creation of highly defined and intricate scaffold architectures. The study found that the Gel/HAP/gly ink could be fabricated with tunable properties to obtain scaffolds with custom pore design, and the presence of HAP significantly enhanced the osteogenic differentiation of encapsulated hASCs demonstrated by the expression levels of COL-I and RUNX2 genes. Mechanical testing showed that the scaffolds possessed adequate strength for potential in vivo applications in hard tissue regeneration. In another study, Allen et al. [182] added GelMa to the Gel/HAP complex so as to leverage the structural and biological benefits of each component. GelMA provides a robust, UV-crosslinkable matrix, gelatin supports cell adhesion and viability, and HAP promotes osteogenic differentiation and improves mechanical properties. The bioprinting was conducted after mixing the composite biomaterial with MC3T3-E1 cells (1.5 × 10^6^ cells/mL), using an extrusion-based method, and the results demonstrated that the addition of HAP significantly reduced the swelling of the hydrogels and improved their resistance to enzymatic degradation, both properties being dependent on the HAP content. Furthermore, the bioink supported high cell viability (over 76 %) and cell proliferation after 28 days, with increased expression of osteogenic markers such as ALP, BMP-7, and OCN, indicating enhanced osteogenic differentiation and mineralization with increasing HAP concentration (up to 20 mg/mL). Furthermore, Gharibshahian et al. [183] introduced a complex ink composition involving PCL, β-TCP, nano-hydroxyapatite (nHAP), and magnesium oxide (MgO), coated with gelatin and loaded with rosuvastatin (RSV). The study aimed to enhance both angiogenic and osteogenic properties of the scaffolds for bone regeneration. The bioprinting process employed was an extrusion technique with subsequent oxygen plasma treatment to improve the surface properties and RSV loading. The scaffolds demonstrated that the mechanical properties of the scaffold, i.e., compressive strength and elastic modulus, were improved after gelatin coating, but RSV loading leads to a decrease in both properties. The authors measured the physical properties of the scaffold before and after degradation for 30 days and reported a compressive strength of 30.60 ± 0.9 MPa for PCL/β-TCP/nHAP/MgO/Gel, which decreased to 27.32 ± 0.76 MPa after degradation, while the elastic modulus decreased from 63 ± 1.43 MPa to 57.61 ± 2.11 MPa. In the case of PCL/β-TCP/nHAP/MgO/Gel-RSV, the compressive strength decreased from 24.62 ± 2.05 MPa to 20.29 ± 1.03 MPa, while the elastic modulus decreased from 48.44 ± 1.02 to 42.05 ± 0.35 MPa following degradation. Moreover, it was demonstrated that introducing gelatin and RSV into the system, improves degradation rate, cell viability (approximately 125 %), ALP activity, and gene expression of COL-I, BMP-2, and OCN, properties that are highly important for osteogenesis. In vivo studies on Wistar rat calvaria defects exhibited significantly improved bone regeneration, filling approximately 86 % of the defect area within three months. Moreover, the results demonstrated high protein production and angiogenesis, as well as an increase in the expression of COL-I, OCN, and VEGF, supporting the high potential of this formulation for bone regeneration.

Gelatin-based bioinks incorporating cellulose have shown great promise in BTE due to their favorable properties, such as biocompatibility, biodegradability, and support for cell proliferation and differentiation. Two recent studies explore different compositions and methods of using cellulose in gelatin-based formulations. Firstly, Cernencu et al. [184] developed a printable mixture composed of a gelatin/pectin polymeric matrix reinforced with GO and oxidized nanocellulose fibers. This composite ink aimed to enhance the mechanical strength, stability, and osteogenic potential of the scaffolds, and it was formulated with GelMA, pectin methacrylate (PeMA), CNF, and varying concentrations of GO (0.25 %, 0.5 %, and 1 % w/w relative to the polymer content). The 3D printing was performed using a microvalve-based bioprinter, which allowed for precise control over the printing process and ensured high-fidelity scaffold structures. The results indicated that the incorporation of GO improved the elastic modulus, printability, and scaffold fidelity, and the optimal GO concentration was found to be 0.5 %, which provided the best balance between mechanical properties and printability, while also being under the cytotoxic threshold [185]. The GO addition to the GelMA/PeMA/CNF system also increased slightly the ink viscosity and improved the enzymatic degradation of the scaffold, evaluated after 7 days. The printing formulation demonstrated good cell viability and proliferation, with the best results obtained using 1 % GO, as was the case for the cytotoxicity test, where this formulation exhibited the lowest levels of lactate dehydrogenase (LDH) after 6 days of cultivation. Secondly, Wang et al. [186] focused on developing a bioink composed of gelatin and maleic acid (MA)-modified bacterial cellulose (MA/BC/Gel) for 3D bioprinting of bone scaffolds. The authors report that the modification with maleic acid enhanced the dispersibility and homogeneity of bacterial cellulose within the ink. Moreover, the formulation with the best results was obtained from 1:30 MA:BC and showed good printability and mechanical properties, resulting in an ink that demonstrates good rheological properties and compression modulus suitable for BTE. The in vitro tests showed that the MA/BC/Gel formulation possessed excellent biocompatibility, significantly enhanced cell viability compared to the control, showing no cytotoxic effects, and promoting osteoblast proliferation. At the same time, it showed higher ALP activity compared to BC, indicating enhanced osteogenic differentiation, and cells treated with the composite exhibited higher messenger ribonucleic acid (mRNA) levels of ALP, COL-I, and RUNX2 genes, highlighting its potential for bone regeneration applications.

Gelatin-based bioinks incorporating decellularized bone (DB) particles have shown promising results for bone tissue engineering, as illustrated by the studies from Kara et al. [129,187]. In the 2022 study conducted by this group [187], the ink was composed of gelatin and DB particles with diameters of approximately 100 μm, obtained from rabbit femur. The DB particles were mixed with gelatin in different concentrations (1 %, 3 %, and 5 % DB) to form a composite scaffold. The ink formulations were crosslinked using microbial transglutaminase (mTG) enzyme, followed by freeze-drying to obtain porous structures, and the 3D printing was performed using an extrusion-based method. The scaffolds were characterized for their morphological, mechanical, and chemical properties, and their cytocompatibility was assessed using MC3T3-E1 mouse pre-osteoblast cells cultured for 21 days. The results demonstrated that the DB-reinforced gelatin hydrogels had a homogenous distribution, enhanced bioactivity, and cytocompatibility. The Young's modulus, swelling ratio, and degradation rate of the scaffolds improved with increasing DB content. No cytotoxic effects were observed on any of the formulations, and it was shown that the incorporation of DB improved cell viability, proliferation, and attachment, indicating that the scaffolds were mechanically robust and appropriate for BTE applications. The authors further explored the incorporation of DB particles into gelatin bioinks in a more recent study [129], aiming to develop minimalistic bioink formulations using different sizes (≤45 μm and ≤100 μm) and concentrations (1 %, 5 %, 10 % wt%) of DB particles mixed with MC3T3-E1 cells (5 × 10^6^ cells/mL). The bioinks were evaluated for their printability and rheological properties, and it was demonstrated that the viscosity of the bioink increased with the addition of DB particles and the printability was generally good, the optimal formulation being the one containing 5 % DB (45 μm). The study used both MC3T3-E1 and hMSCs to assess cell viability, proliferation, and osteogenic differentiation. It was shown that the incorporation of DB particles significantly enhanced cell viability and proliferation, especially in the Gel/10 % DB bioink, which showed cell proliferation inside the scaffold (Fig. 3 B), attributed to the natural collagen and HAP content of the DB particles. ALP activity was notably increased, indicating enhanced osteogenic potential, especially in the bioformulation containing Gel/10 % DB (45 μm). Fluorescence microscopy revealed pronounced cell-material interactions and cell attachment within the constructs, further supporting the potential of these bioinks for clinical translation in BTE.

These studies collectively demonstrate that gelatin-based bioinks, enhanced with various bioactive and structural additives, provide a promising foundation for developing functional and clinically applicable scaffolds for bone tissue regeneration. It was demonstrated that incorporating these additives into gelatin-based formulations improves their mechanical properties, printability, and biological performance, providing a supportive environment for cell growth and tissue formation, closely mimicking the natural bone matrix. For instance, adding decellularized bone particles has been shown to increase cell viability, proliferation, and osteogenic differentiation, making the bioinks more effective for BTE. The most common 3D printing method was extrusion-based in the studies presented. These findings open the door to further refinement and potential clinical applications in treating bone defects and improving outcomes in orthopedic and reconstructive surgeries.

#### 3.2.4 Collagen-based (bio)inks

Collagen-based inks and bioinks for BTE have shown significant potential due to their excellent biocompatibility, biodegradability, and ability to mimic the natural ECM. These inks range from simple collagen formulations to complex composites with various enhancements to improve their mechanical properties, printability, and biological functionality.

The simplest collagen inks consist of pure collagen, which offers high biocompatibility and biodegradability. However, pure collagen inks suffer from poor mechanical properties and rapid degradation, limiting their application in load-bearing bone tissue engineering. For example, Snider et al. [42] explored the fabrication of liquid collagen inks for 3D printing, emphasizing the need for crosslinking agents to enhance the stability and structural integrity of the printed scaffolds, while demonstrating that the collagen-based 3D scaffolds support cell viability and proliferation. Particularly, the authors investigated two printing methods, collagen crosslinking with several concentrations of N-(3-dimethylaminopropyl)-N′-ethylcarbodiimide (EDC) - N-hydroxysuccinimide (NHS) and several concentrations of genipin, as well as the conjugation with gold nanoparticles (AuNPs). The study shows that liquid collagen can be successfully used as printable formulation not only for BTE, but also for a variety of applications in tissue engineering, by modulating the crosslinking and the conjugation with AuNPs. Moreover, Nayak et al. [188] investigated type 1 bovine collagen scaffolds for tissue engineering applications. This study focused on creating a fibrillar colloidal gel from collagen, which was then 3D printed to form scaffolds with engineered pore architectures. The scaffolds were further chemically crosslinked using EDC followed by lyophilization, further noted CCL. The study found that the crosslinked scaffolds were thermally stable at body temperature, supported cell attachment and proliferation, and showed higher cell viability and ALP expression when compared to the control. However, crosslinking reduced the mechanical strength of the scaffolds (the maximum tensile stress was 2.65 ± 1.89 MPa for CCL and 7.89 ± 1.89 MPa for the control), indicating a trade-off between structural stability and biological functionality.

To address the limitations of pure collagen, collagen-hydrogel composites have been developed. Guo et al. [189] focused on a collagen-HAP scaffold fabricated using a gelation bath for 3D printing. The authors developed the bioink by mixing the Col/HAP hydrogel with BMSCs (2 × 10^6^ cells/mL), and it was shown that the inclusion of HAP enhances the scaffold's mechanical properties and osteoconductivity, mimicking the natural bone composition. The use of a gelatin support bath aids in maintaining the structural integrity of the scaffold during the printing process at room temperature, allowing for precise and stable fabrication of the 3D structures. The study demonstrates that HAP particles are uniformly distributed in the scaffold and that the 3D structure has good mechanical properties, the compressive modulus increasing after scaffold lyophilization, and a high elasticity, the scaffold recovering nearly 100 % of the original shape under maximum strain of 80 %. Moreover, the resulting constructs demonstrated increased cell viability and higher ALP expression compared to the control (Fig. 3 C), while also supporting the proliferation and differentiation of BMSCs, highlighting their potential for effective BTE. In a similar study, Kim et al. [190] developed a composite bioink using collagen and β-TCP mixed with hASCs and human umbilical vein endothelial cells (HUVECs) at a concentration of 2 × 10^7^ cells/mL. The addition of β-TCP aimed to mimic the mineral composition of natural bone, enhancing the osteoinductive properties of the scaffold. The study demonstrated that the collagen/β-TCP bioink has good rheological properties (the yield stress increased with β-TCP content), and the scaffold printed with the bioink containing hASCs/HUVECs supported cell viability, adhesion, and proliferation better than hASC-constructs. Moreover, the scaffold also showed superiority in osteogenic marker expression (ALP, RUNX2, OCN, and OPN) and it demonstrated high potential for bone formation in vivo. The scaffolds were implanted in female C57BL/6 mice, and after 6 weeks it was demonstrated a high fusion rate (90 %), 1.3 higher bone volume over total volume (BV/TV), and higher bone mineral density compared to the control, as well as blood vessel formation and no inflammatory reaction, showing great promise in spinal fusion and BTE applications.

Nagaraj et al. [191] investigated collagen-GelMA hydrogels for their potential in bone tissue engineering. They combined GelMA with two types of collagen (ovine and bovine) in different concentrations to enhance the ink's mechanical properties and printability. The study found that this combination improved the scaffold's stability, and the hybrid hydrogels incorporating 1 % collagen, either bovine or ovine, demonstrated the best balance of printability, mechanical properties, and stability, making them suitable for various tissue engineering applications. Moreover, Suo et al. [47] explored a low-temperature 3D printing method using a composite of collagen and chitosan. This approach focused on maintaining the bioactivity of collagen and improving printability. The low-temperature technique helped retain the functional properties of the collagen while ensuring structural fidelity during printing. The resulting scaffolds prepared with 2 % Col and 2 % Chi showed good mechanical properties, with a high Young's modulus (61.980 ± 10.139 kPa) and the highest tensile strength compared with the other concentrations. The swelling ratio decreased with increasing chitosan proportion, indicating more compact structures, and the degradation rate was lower in comparison with the other formulations (50 % after 24 h). Cell viability was measured at over 97 %, and it was shown that the cells migrated to the bottom of the scaffold. Overall, the results indicate that the combination of collagen and chitosan improves the printability and mechanical properties of the scaffolds, while maintaining high cell viability and controlled degradation rates, making them suitable for tissue engineering applications.

A more complex approach was taken by Heide et al. [192], who combined collagen, tyramine modified hyaluronic acid (THA), and calcium phosphate to create a multifunctional scaffold. This study aimed to leverage the synergistic effects of these components to enhance the bioactivity and mechanical properties of the 3D printed structure. The results showed good cell viability and osteogenic differentiation potential, demonstrated by osteoprotegerin (OPG) secretion, ALP activity (decreased with CaP concentration), and upregulation of integrin binding sialoprotein and matrix metalloproteinases-13 genes. Combined with the mechanical properties (slowed degradation and high compressive modulus) and excellent printability, the Col/THA/CaP formulation is a promising candidate for BTE applications. At the same time, Lan et al. [193] introduced a double-crosslinked bioink containing thiol-modified hyaluronic acid (tHA), photocurable acrylated type I collagen (ColMA), poly(ethylene glycol) diacrylate (PEGDA), and nasal chondrocytes (NC) in a concentration of 1 × 10^7^ cells/mL, specifically designed for cartilage repair but with potential applications in BTE. The double crosslinking technique provided good printability and enhanced mechanical stability, the scaffolds supporting high cell viability post-printing (85 %) and post-crosslinking (83 %). The results also showed that the 3D structure has a high capacity for cartilage ECM production, demonstrated by chondrogenic (ACAN, COL2A1, SOX9) and hypertrophic (COL10A1, RUNX2) gene expression.

Comparing these studies, it is evident that the evolution of collagen-based bioinks has been driven by the need to balance biocompatibility, mechanical strength, and bioactivity. Simple collagen bioinks are limited by their mechanical properties, while hydrogel composites and bioceramic integrations offer improved stability and functionality. Advanced composites incorporating multiple bioactive components provide a synergistic effect, enhancing both the biological and mechanical properties of the scaffolds. These developments pave the way for more effective and customized approaches to bone regeneration and repair, addressing the specific requirements of different BTE applications.

#### 3.2.5 Chitosan-based (bio)inks

Chitosan-based inks have shown significant potential for BTE due to their biocompatibility, biodegradability, and ability to support cell growth [194]. There are studies on complex hydrogels that have the potential to be developed as printable formulations and used in bone tissue regeneration, showing remarkable properties. As such, Coskun et al. [195] developed a chitosan-based bioink incorporating amorphous nHAP, which was prepared using a combination of chitosan (2.3 % w/v) with glycerol phosphate disodium hydrate salt (GP) and sodium hydrogen carbonate (SHC) as crosslinking agents. Different concentrations of nHAp (10 %, 25 %, 40 % w/w) were added to enhance the bioink's properties. The printable formulation demonstrated shear-thinning behavior, making it suitable for extrusion-based 3D printing. Optimal printability was achieved with a 27 G nozzle at pressures between 50 and 70 kPa and speeds of 4–11 mm/s. High cell viability (over 90 %) was observed with pre-osteoblastic MC3T3-E1 (2 × 10^6^ cells/mL) post-printing, indicating the bioink's suitability for bone tissue applications. It was shown that the presence of nHAp improved the mechanical properties and printability, while maintaining high cell viability, making them suitable for BTE applications. Maturavongsadit et al. [196] developed a composite bioink combining chitosan, CNC, and MC3T3-E1 cells (5 × 10^6^ cells/mL) to enhance the mechanical properties and the printability of the scaffold, obtained by the extrusion method. The results showed that the Chi/CNC bioink had improved rheological properties, allowing for the fabrication of complex 3D structures with high fidelity, while the mechanical testing indicated a significant increase in compression modulus (1.5-fold) compared to pure chitosan bioinks. Cell culture studies demonstrated high cell viability (over 90 %) and proliferation, suggesting its potential for meniscus repair and BTE.

More recently, Bharadwaj et al. [197] developed a thermosensitive bioink incorporating chitosan and gelatin self-assembled nanofibrous aggregates (Gel-SNA) combined with nHAP. The optimal composition identified contained 2 % chitosan, 1 % Gel-SNA, 10 % nHAP, and 5.6 % β-glycerophosphate (βGP), offering enhanced printability, good mechanical properties, and high cell viability (over 88 %). This printable formulation demonstrated a low water uptake capacity (2.5 %), an elastic modulus of 15.5 kPa, and low degradation rate (three weeks in the presence of lysozymes). The composite scaffold showed osteogenic potential, demonstrated by high ALP activity (36.8 units/ml after 14 days in culture) and significant calcium deposition (Fig. 3 D) after 14 days in osteogenic media (28.76 ± 1.78 μg/mg).

Another recent study conducted by Chang et al. [198] explored the use of methacrylated glycol chitosan (Me/gly/Chi) as a printable formulation for BTE. The Me/gly/Chi bioink (1 × 10^6^ MG-63 cells/mL), prepared at a 3 % concentration with riboflavin (12 μM) as the photoinitiator, was optimized for 3D printing using an extrusion-based bioprinter with a 26 G nozzle and a pneumatic pressure of 120 kPa. The ink was cured under visible light for 30–90 s, ensuring high printability and structural integrity. The formulation photo-cured for 70 s demonstrated excellent mechanical properties, with a compressive modulus of 25.7 kPa, a low swelling ratio, and a low degradation rate (28.4 % remained after 75 days). At the same time, the scaffold maintained high cell viability (>92 %) and proliferation (>96 %) of MG-63 cells. Additionally, it exhibited significant osteogenic differentiation, confirmed by elevated ALP activity and calcium deposition after 7 days. The proposed ink also showed low cytotoxicity and hemolysis levels (<2 %), making it suitable for creating patient-specific scaffolds for bone regeneration.

In the field of BTE, lab-made printable formulations have demonstrated significant promise in advancing regenerative medicine. These formulations, often composed of synthetic polymers, ceramics, and natural polymers like agarose, alginate, gelatin, collagen, and chitosan, are designed to replicate the complex structure and functionality of natural bone tissue. The incorporation of cells into these inks further enhances their potential by promoting cell growth and tissue regeneration. Despite the challenges associated with regulatory approval and the need for extensive clinical trials, ongoing research and advancements in 3D printing technology are paving the way for these innovative solutions to become standardized and eventually commercially available. The presented chapter highlights several formulations, each showcasing different combinations of materials tailored to improve mechanical properties, printability, and biological performance, ultimately offering personalized solutions for bone repair treatments.

## 4 Challenges and future perspectives in bioprinting for BTE

The fabrication of bioinks for 3D bioprinting presents several challenges, particularly the need for standardization in order to guarantee consistent and dependable outcomes in a variety of applications. Variability in bioink preparation, which frequently results from manual handling by various operators, is a common and important issue because printability, cell viability, and bioink uniformity may all suffer as a result. Studies reveal that even small changes in variables such as mixing speed and number of mixing exchanges can have a big impact on the properties of the bioink and, in turn, the quality of the structures that are bioprinted [61,199,200]. Another challenge is represented by the intricacy of bioink formulations, which frequently involve complex combinations of hydrogels and cells that must maintain both their mechanical strength and biological functionality throughout and after the printing process [199,201]. The development of bioinks will be influenced by the establishment of automated systems and standardized protocols that minimize human error and variability, thereby improving the reproducibility of bioprinted tissues. Compared to manual methods, automated mixing devices have demonstrated the potential to improve bioink consistency by providing better control over mixing parameters, resulting in more consistent and dependable bioinks [200]. The advancement of 3D bioprinting from experimental research to scalable biomedical applications will depend on this move toward standardization.

Another aspect that is essential for 3D bioprinting is the resolution, as it directly affects the fidelity and functionality of the printed constructs, which is necessary to replicate the intricate microarchitectures of biological tissues [202]. The exact deposition of bioinks at the cellular and subcellular levels is made possible by high-resolution bioprinting techniques, and this is crucial for building structures that closely resemble the ECM and other tissue components [41]. This level of accuracy is essential for both preserving cell viability and regulating cellular behavior, which is influenced by chemical and physical cues from the microenvironment; these behaviors include cell migration, differentiation, and alignment [203]. Consequently, high-definition bioprinting is becoming an essential tool in tissue engineering and regenerative medicine, as it can produce feature sizes smaller than 50 μm [204]. However, there are many obstacles in the way of achieving and sustaining high resolution in 3D bioprinting, especially when it comes to the creation and design of bioinks. Finding a balance between the resolution, printing volume, and speed is one of the biggest challenges. Slower printing processes brought on by high-resolution techniques can negatively influence cell viability during lengthy fabrication times [205]. Furthermore, it is difficult to develop bioinks that are both biocompatible and appropriate for high-resolution printing. For these bioinks to be precisely deposited without clogging printer nozzles or losing their structural integrity, they must have the proper viscosity, crosslinking qualities, and mechanical strength [204]. The development of novel smart formulations responsive to stimuli that facilitate multimaterial printing and that are compatible with hybrid bioprinting devices will be necessary as the field develops in order to create more intricate and functional tissue constructs. Overcoming these obstacles is vital for the future of bioprinting, especially in the development of new bioinks that can maintain high resolution and facilitate quicker and more scalable production of tissue-engineered products.

**Fig. 4:**
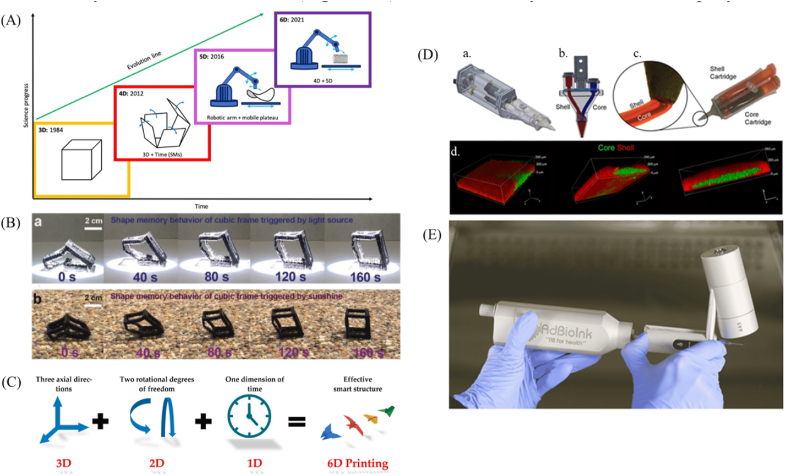
(A) Schematic representation of the evolution from 3D printing to 6D printing [220] (CC BY 4.0); (B) 4D printed constructs showing the shape recovery in time under (a) 87 mW cm^−2^ of light source and (b) 76 mW cm^−2^ of sunshine. Reprinted from Ref. [221] with permission from WILEY. (C) Schematic representation of the 6D printing method [212]; (D) Core/shell 3D printing by co-axial extrusion: (a) diagram of the co-axial handheld 3D printer; (b) diagram of the co-axial nozzle; (c) image of the cartridges designed for core and shell loading in the printer; (d) Example of 3D rendered confocal images of a printed sample labeled with fluorescent beads [222] (CC BY 4.0). (E) BioPen-X device) commercialized by AdBioInk [223].

Nevertheless, the future of bioprinting in the BTE field is rapidly advancing with the introduction of multi-dimensional printing technologies, specifically 4D, 5D, and emerging 6D bioprinting (Fig. 4 A). These advanced techniques promise to overcome current limitations and provide innovative solutions for complex medical challenges. 4D bioprinting extends traditional 3D printing by incorporating the dimension of time, allowing printed structures to transform shape (Fig. 4 B) or function in response to external stimuli such as temperature, humidity, or light [206,207]. This dynamic capability is achieved using smart materials, such as shape memory polymers and hydrogels, which enable the printed constructs to change shape post-fabrication [208]. In BTE, 4D bioprinting can create adaptive scaffolds that evolve with the patient's healing process, providing tailored mechanical support and facilitating tissue regeneration.

5D bioprinting enhances the complexity and structural integrity of printed objects by using five axes of movement during the printing process [209]. This technology can enable the creation of more intricate and robust curved structures that better mimic the natural morphology of human bones [210]. The ability to print from multiple angles improves the mechanical properties of the constructs, making them stronger and more resilient compared to traditional 3D printed objects [211]. In orthopedics, 5D printing can be particularly beneficial for producing patient-specific implants and prosthetics that require precise anatomical conformity and high durability.

The latest advancement, 6D bioprinting, combines the principles of 4D and 5D printing using multiple axes (Fig. 4 C). It produces objects that not only have complex, curved geometries but also possess the ability to change shape or properties over time in response to environmental stimuli [212]. This approach uses smart materials within a five-axis printing framework to create highly functional and adaptive medical devices. For instance, 6D bioprinting could develop smart orthopedic casts that adjust tension and fit in response to swelling or movement, enhancing patient comfort and treatment outcomes [124,213]. The integration of such adaptive features holds significant promise for pediatric orthopedics, where growing bones require dynamic support systems.

Moreover, the future of medicine will most likely be changed for the better with the use of miniaturized devices like the Biopen, which is a cutting-edge, portable bioprinting device (Fig. 4 D) designed to facilitate the direct deposition of bioinks in surgical settings, particularly for applications in BTE such as bone regeneration and cartilage repair [214,215]. This handheld tool allows surgeons to “draw” directly onto bone defects with cell-laden hydrogels, enabling real-time, in situ bioprinting of customized scaffolds [216]. The development of the Biopen represents a significant advancement in 3D bioprinting technology, combining the precision of additive manufacturing with the flexibility and immediacy required in clinical environments and point-of-care applications [217]. One of the main advantages of the Biopen is its ability to deliver bioinks that promote bone regeneration directly at the site of injury or defect. The device typically uses extrusion-based bioprinting technology, which is well-suited for producing cell-laden hydrogel constructs [218]. The extrusion system within the Biopen is designed to be compatible with a range of bioinks, including those made from materials like alginate, gelatin, and chitosan, which have been shown to support osteogenic differentiation and bone tissue formation [216].

The Biopen's portability and ease of use make it particularly valuable for orthopedic surgeries, where precise application of bioinks can be critical for successful outcomes. The ability to deposit cells and biomaterials directly into the defect site allows for the creation of complex, 3D structures that conform perfectly to the patient's anatomy [219]. This level of customization is crucial for ensuring that the bioprinted scaffold integrates well with the surrounding tissue and provides the necessary mechanical support as new bone tissue forms. This kind of device is already commercially available as BioPen-X (Fig. 4 E), which is sold by the AdBioInk company.

The evolution of bioprinting technologies from 3D to 6D, as well as novel bioprinting approaches, represents a significant leap in the capability to engineer complex, adaptive, and patient-specific medical solutions. These advancements hold immense potential for bone tissue engineering, offering tailored and dynamic treatments that can significantly improve patient outcomes. As these technologies mature, they will likely become integral to the future of personalized medicine and regenerative orthopedics.

## 5 Conclusions

Bioprinting and bioinks for BTE have emerged as transformative technologies with the potential to revolutionize regenerative medicine and orthopedics. This review has delved into various bioprinting methods, including extrusion-based, inkjet-based, laser-assisted, light-based, and hybrid technologies. Each technique offers unique advantages and limitations, shaping their applicability in tissue engineering. Extrusion-based bioprinting stands out for its ability to print high-viscosity materials and create complex 3D structures. Inkjet bioprinting is notable for its precision and speed, while laser-assisted methods provide high resolution and cell viability. Light-based technologies, such as stereolithography, offer excellent accuracy and surface finish, and hybrid approaches combine multiple methods to optimize printing outcomes.

The review also explores the range of available commercial inks specifically designed for BTE applications. These printable mixtures, often composed of biocompatible materials like hydrogels, ceramics, and composites, are tailored to support osteogenesis and ensure structural integrity. Despite the progress in commercial inks, lab-made formulations continue to play a crucial role in advancing the field. Custom inks developed in research settings enable the exploration of new materials standardization and bioactive compounds, providing insights that drive innovation.

Furthermore, the bioprinting market for BTE is expanding rapidly, fueled by technological advancements and increasing clinical demand. Companies are investing in the development of new bioprinters and bioinks, aiming to create more effective and scalable solutions for bone regeneration. The market growth is also driven by the integration of bioprinting technologies with digital health and personalized medicine, offering customized implants and treatments tailored to individual patient needs.

Looking ahead, the future perspectives of bioprinting and bioinks in this field are promising, as ongoing research is expected to yield bioinks with enhanced mechanical properties, biocompatibility, and bioactivity. The development of multi-material bioprinting and the incorporation of growth factors and stem cells into printable mixtures will likely improve the functionality and integration of printed bone tissues. Additionally, advancements in 4D, 5D, and 6D printing, along with computational modeling and imaging technologies, will enhance the precision and reproducibility of printed constructs.

While significant challenges remain, the field of bioprinting in bone tissue engineering holds great potential to transform the landscape of regenerative medicine. Continuous interdisciplinary collaboration and innovation are essential to overcome current limitations and unlock the full therapeutic potential of these cutting-edge technologies. As research and development efforts progress, bioprinting is poised to make a profound impact on the treatment of bone injuries and diseases, ultimately improving patient outcomes and quality of life.

## CRediT authorship contribution statement

**Elena Alina Chiticaru:** Writing – original draft, Investigation, Conceptualization. **Mariana Ioniță:** Writing – review & editing, Supervision, Project administration, Funding acquisition, Conceptualization.

## Statement of significance

This review explores the latest advances in the formulation of inks and bioinks for bone tissue engineering (BTE) applications. It highlights both commercial and lab-made state-of-the-art printable formulations, especially based on natural polymers like agarose, alginate, gelatin, and collagen, and examines the strengths and limitations of various bioprinting technologies. By addressing novel printing methods, including emerging 4D, 5D, and 6D techniques, the review underlines their potential to revolutionize personalized medicine. This work is significant for advancing BTE by improving fabrication processes and clinical outcomes, making it relevant to both researchers and practitioners in regenerative medicine and biofabrication.

## Declaration of generative AI and AI-assisted technologies in the writing process

Statement: During the preparation of this work the authors used ChatGPT by OpenAI in order to improve the readability and language of the manuscript. After using this tool, the authors reviewed and edited the content as needed and take full responsibility for the content of the published article.

## Declaration of competing interest

The authors declare that they have no known competing financial interests or personal relationships that could have appeared to influence the work reported in this paper.

## Data Availability

No data was used for the research described in the article.
